# Nightmares and psychiatric symptoms: A systematic review of longitudinal, experimental, and clinical trial studies

**DOI:** 10.1016/j.cpr.2022.102241

**Published:** 2023-03

**Authors:** Bryony Sheaves, Stephanie Rek, Daniel Freeman

**Affiliations:** a Department of Psychiatry, University of Oxford, Warneford Hospital, Oxford OX3 7JX, United Kingdom; b Oxford Health NHS Foundation Trust, Warneford Hospital, Oxford, OX3 7JX, United Kingdom; c Department of Psychiatry and Psychotherapy, LMU University Hospital Munich, Munich, Germany; d International Max Planck Research School for Translational Psychiatry (IMPRS-TP), Munich, Germany

**Keywords:** Nightmares, Depression, Anxiety, Post-traumatic stress disorder, Psychosis, Suicide

## Abstract

Nightmares occur across a wide range of psychiatric disorders, but outside of PTSD presentations are infrequently considered a treatment priority. We aimed to assess evidence for a contributory causal role of nightmares to the occurrence of psychiatric disorders, and vice versa. A systematic review was conducted of longitudinal, experimental, and clinical trial studies. Twenty-four longitudinal, sixteen trials, and no experimental studies were identified. Methodological shortcomings were common, especially the use of single-item nightmare assessment. Thirty-five studies assessed the path from nightmares to psychiatric symptoms. Depression (*n* = 10 studies), PTSD (n = 10) and anxiety (*n* = 5) were the most commonly assessed outcomes in trials. Most were not designed to assess the effect of nightmare treatment on psychiatric symptoms. Treating nightmares led to moderate reductions in PTSD and depression, small to moderate reductions in anxiety, and potentially moderate reductions in paranoia. Nightmares increased the risk of later suicide outcomes (*n* = 10), but two small pilot trials indicated that treating nightmares might potentially prevent recovery of suicidal ideation. PTSD treatment led to large reductions in trauma-related nightmares (*n* = 3). The limited literature suggests that treating nightmares may be one route to lessening threat-based disorders in particular, suggestive of a causal relationship. Overall, however, nightmares in most disorders are greatly understudied.

## Highlights

• Treating nightmares improves threat-based disorders (PTSD, anxiety and paranoia).
• Depressive symptoms are alleviated by nightmare treatments.
• Nightmares are a risk factor for later PTSD and suicide.
• PTSD treatment leads to large reductions in trauma-related nightmares.
• Treating nightmares in a clinical sample with depression requires testing.

## 1 Introduction

*“And dreams in their development have breath,*

*And tears, and tortures, and the touch of joy;*

*They leave a weight upon our waking thoughts,*

*They take a weight from off waking toils”*

‘The Dream’, Lord Byron

The content of dreams has fascinated writers, philosophers, and physicians for centuries. In the fifth century BCE the Greek physician Hippocrates considered that nightmares had importance: *‘he who has learnt aright about the signs that come in sleep will find that they have an important influence upon all things’*. The observation of ‘*nightmares and sleeplessness’* in psychiatric patients was noted in Kraeplin's (1919) early descriptions of what came to be known as schizophrenia. Yet despite this longstanding interest in dream content and the recognition that dreams might be an indicator of psychological health, problematic dreams - nightmares - are rarely assessed in patients attending psychiatric services and almost never directly treated. In this paper, the ‘weight upon our waking thoughts’ - the potential impact of nightmares on a range of mental health disorders - is reviewed.

Nightmares involve vivid and distressing mental imagery which wake the individual, thereby limiting restorative sleep. The most common themes of nightmares include physical aggression, failure, or helplessness (Robert & Zadra, 2014). The point prevalence of frequent nightmares lies around 3–7% in the general population (Janson et al., 1995; Sandman et al., 2013), with estimates substantially higher for psychiatric populations. For individuals diagnosed with post-traumatic stress disorder (PTSD), trauma-related nightmares are a symptom of the disorder, and occur in two thirds of patients (Swart, Van Schagen, Lancee, & Van Den Bout, 2013). Hence in clinical services nightmares have typically been conceptualised as a reaction to trauma. But nightmares can also occur outside of PTSD. Idiopathic nightmares (i.e. those not attributable to trauma) typically begin in childhood, are attributable to moderate genetic influence, and individuals who experience frequent childhood nightmares have three times greater odds of a serious psychiatric disorder in adulthood compared with those who never experience childhood nightmares (Hublin, Kaprio, Partinen, & Koskenvuo, 1999). The mechanisms underlying idiopathic nightmares are likely to be independent of trauma, but the causes remain greatly under-researched.

Researchers make a distinction between idiopathic and posttraumatic nightmares, but in mental health services nightmares are primarily only assessed as part of PTSD. This means that nightmares occurring alongside other mental health disorders go largely undetected and untreated (Nadorff, Nadorff, & Germain, 2015; Reeve, Sheaves, & Freeman, 2018). Yet nightmares are prevalent for patients experiencing other diagnoses too. For example, half of those diagnosed with a psychotic disorder screen positive for nightmare disorder (Reeve, Sheaves, & Freeman, 2018; Sheaves, Onwumere, Keen, Stahl, & Kuipers, 2015). Yet far fewer patients with psychosis screen positive for PTSD at any one time (16%; De Bont et al., 2015). Looking across disorders, nightmares are a problem for at least a third of those with mood disorders (Sjöström, Wærn, & Hetta, 2007; Swart et al., 2013), over half of individuals with dissociative disorders (Agargun et al., 2003), and just under a fifth of those with anxiety disorders (Swart et al., 2013). Two thirds of psychiatric inpatients admitted after a suicide attempt complain of nightmares (Sjöström et al., 2007). Whilst it is likely that some cases are attributable to co-occurring PTSD, it is also plausible that a proportion occur outside of a reaction to trauma, are diagnosable as nightmare disorder, and require independent clinical attention.

Network approaches to psychopathology provide an alternative framework to explain why nightmares might be less specific to PTSD than described within diagnostic manuals. Rather than a latent variable causing a specific constellation of symptoms (e.g. PTSD causing nightmares, flashbacks, and avoidance of trauma reminders), the implication of network approaches is that clinical disorders arise from the interplay between symptoms (Borsboom & Cramer, 2013). Feedback loops between specific mental health experiences occur (e.g. nightmares might leave someone feeling anxious, and in turn the hyperarousal might increase the chance of having subsequent nightmares), and these loops account for the high rate of comorbidity between psychiatric disorders (Borsboom & Cramer, 2013; Borsboom, Cramer, Schmittmann, Epskamp, & Waldorp, 2011; Cramer, Waldorp, Van Der Maas, & Borsboom, 2010). The clinical implication of this account is that treating individual symptoms that have strong causal connections within a network are a greater priority (Borsboom & Cramer, 2013; Freeman, Sheaves, Waite, Harvey, & Harrison, 2020). Here, the treatment of individual symptoms that bridge and causally impact other clusters of symptoms are a priority for intervention because they could bring about the largest effect on the whole network of psychopathology. As an example, network approaches have recently demonstrated that dissociation influences a range of other mental health problems, and should therefore be given greater clinical priority outside of the context of PTSD (Černis, Evans, Ehlers, & Freeman, 2021). We have also recently highlighted that the treatment of sleep disruption is a clinical priority (Freeman et al., 2020). Insomnia, for example, shows a bidirectional relationship with major mental health problems, with typically the strongest path from insomnia to psychiatric problems. In this paper we consider whether the specific sleep problem of nightmares may be a contributory factor for the occurrence of a range of psychiatric symptoms, and hence may be of greater importance than currently recognised.

### 1.1 From nightmares to psychiatric symptoms

*“When something gory happens [in the nightmare], I see it in real detail and then when I wake up that sort've emotion… it doesn't go away, it plays on my mind all day”*

‘Ben’ – an outpatient experiencing psychosis and nightmares

Nightmares are vivid, immersive experiences that directly induce negative affective states. The brain responds as if seeing and feeling the events that occur during dreaming. There is evidence of hyperactivation of the sensory cortices, visual association areas, and limbic structures (Desseilles, Dang-Vu, Sterpenich, & Schwartz, 2011; Maquet et al., 1996). There are marked increases in autonomic nervous system activity during REM sleep when a nightmare occurs (Paul, Alpers, Reinhard, & Schredl, 2019), resulting in physical symptoms of anxiety (e.g. sweating and shortness of breath) (Böckermann, Gieselmann, & Pietrowsky, 2014). One feature of nightmares which may make them a particularly potent form of mental imagery is the lack of control over events during dreaming, and often uncritical acceptance of bizarre, implausible and distressing events as real (Desseilles et al., 2011; Muzur, Pace-Schott, & Hobson, 2002). Whilst this dissipates to varying degrees upon awakening, for some people it can result in persistent distress. Although the content of nightmares is not real, they can act as real stressors that trigger daytime emotional difficulties, as has been found for other types of stressors and the subsequent development of emotional disorders (Gulley, Hankin, & Young, 2016; Hammen, Henry, & Daley, 2000). When nightmares occur frequently, they may trigger more persistent negative affect, which is a core symptom of affective disorders.

We propose that once negative affect is triggered by a nightmare, nightmare-related negative appraisals, and cognitive styles such as worry and rumination, exacerbate it. The clinical example at the start of the section ‘Ben’ describes ruminative thinking (“*it plays on my mind*”) which likely prolongs the negative affect. The function of this thinking style is often described by patients as a means of making sense of the nightmare. Yet this can result in catastrophic interpretations which exacerbate distress (Gieselmann, Elberich, Mathes, & Pietrowsky, 2020). In our clinical experience with clients experiencing psychotic symptoms, typical cognitions can include “the nightmares mean I'm a bad person” (negative self-beliefs), “they are a sign of things that will happen in the future” (beliefs about the world and the future) or “it's my persecutor planting them in my mind” (beliefs about others). These can be associated with unhelpful behavioural responses, for example avoidance of people or events in an effort to avoid events depicted in nightmares. Negative beliefs about the self underlie many psychiatric disorders, but particularly depression. Worries about the future can trigger anxiety and paranoia and negative beliefs about others may underlie paranoia or social anxiety concerns. Of course it is likely that those with a significant problem with nightmares already experience negative core beliefs. Yet nightmares may activate and hence strengthen them.

A separate route linking nightmares with psychiatric symptoms is via destabilisation of the sleep-wake cycle. Nightmares directly interrupt restorative sleep and can trigger a difficulty getting back to sleep (insomnia symptoms). Patients experiencing nightmares also commonly report a fear of sleep (Krakow, Tandberg, Scriggins, & Barey, 1995), which delays sleep onset via increased hyper-arousal. Sleep onset or maintenance insomnia is therefore commonly co-morbid with nightmares (Reeve, Sheaves, & Freeman, 2018), and recent research has shown that insomnia is causally related to a range of mental health problems including depression, anxiety, paranoia and hallucinations (Freeman et al., 2017; Freeman et al., 2020; Reeve, Emsley, Sheaves, & Freeman, 2017). Whilst sleep loss has detrimental effects on mental health (Reeve et al., 2017), the fragmentation of sleep even without any change in total sleep time, also has negative consequences for next day mood and sleepiness (Martin, Engleman, Deary, & Douglas, 1996).

### 1.2 From psychiatric symptoms to nightmares

*“obviously the whole being told that your children are going to be taken away from you [by voices] and then dreaming that your children are going to be taken away from you, that's obviously linked in some way.”*

‘Amy’ – an outpatient experiencing psychosis and nightmares

Nightmares typically occur within rapid eye movement (REM) sleep. Whilst dream recall can also occur within non-REM sleep, dreaming is less prominent, vivid and detailed than in REM sleep (Germain, 2013). Those experiencing psychiatric symptoms could be more vulnerable to nightmares owing to a greater burden on the REM sleep system, due to more frequent and distressing daytime experiences that require processing. The REM sleep emotional homeostasis hypothesis asserts that REM sleep (in which the majority of dreaming occurs) functions to strip away the emotional charge from the previous day's affective experiences whilst consolidating encoding of episodic events (Goldstein & Walker, 2014). Patients commonly describe the content and emotion of distressing daytime experiences as being reactivated in nightmares, which is demonstrated by the clinical example (Amy) at the start of the section. It is possible that nightmares represent a dysfunction of the REM sleep system whereby ineffective processing of emotional memories during REM sleep results in a repeat attempt the subsequent night, and if the process fails again, subsequent nightmares ensue (van der Helm & Walker, 2009). Whilst REM sleep has been linked experimentally with the consolidation of fear extinction memory (for a review, see: Pace-schott, Germain, & Milad, 2015), the prediction that nightmares reflect a dysfunction in this system requires experimental testing. What has been shown is that idiopathic nightmare sufferers experience a lower propensity for REM sleep when compared to healthy controls (Nielsen et al., 2010), and acute stress exposure lowers late night REM density (Germain, Buysse, Ombao, Kupfer, & Hall, 2003).

The link between daytime symptoms and the occurrence of nightmares may be accentuated for people experiencing dissociation. Dissociative experiences have traditionally been considered a consequence of PTSD, but recent research highlights connections with other mental health symptoms too Cernis et al. (2021). A particularly high prevalence of nightmares has been found in dissociative disorders (Agargun et al., 2003). REM dependent emotional processing may be a particular problem for those who have the tendency to manage very strong waking emotion through disconnecting from the negative affect via a detachment type of dissociation. Whilst detachment may provide short term alleviation of distress (Holmes et al., 2005), it negates the opportunity to process the emotion prior to sleep, potentially placing greater burden on the REM sleep system. The neural mechanisms which underpin such psychological escape in the daytime (frontal inhibition of the limbic system) and provide the psychological ‘braking system’ for emotions are also deactivated during REM sleep (Desseilles et al., 2011). This means that psychological escape is not possible during dreaming, resulting in an emotionally intense nightmare. There is some support for this theory: Rek, Sheaves, and Freeman (2017) report an association between depersonalisation (one part of detachment type dissociation) and both the occurrence and severity of nightmares, whilst controlling for PTSD symptoms and negative affect.

### 1.3 Alternative hypothesis: nightmares are not causally related to psychiatric symptoms

It is plausible that nightmares are not causally related to other mental health problems, but instead share similar causes (see Table 1 for a summary). For example, there is a genetic correlation between schizophrenia liability and the risk of childhood nightmares (Reed, Jones, Hemani, Zammit, & Davis, 2019). It is also possible that the co-occurrence of nightmares and psychiatric disorders is attributable to confounding effects, a key candidate being medications. Nightmares can be triggered by medications taken for psychiatric symptoms. Agents exerting pharmacological effects on dopamine, serotonin and norepinephrine for example have been implicated in the occurrence of nightmares, owing to their effect on sleep architecture (Li, Lam, Yu, Zhang, & Wing, 2010; Pagel & Helfter, 2003). However, the prevalence of patients reporting nightmares as a side effect across these medication types remains very low. In a review of clinical trials testing psychiatric medications, less than 5% of patients in the active arm of any of the medication trials reported problematic nightmares (Pagel & Helfter, 2003). Hence they are unlikely to fully account for the half of patients with psychosis, third of those with mood disorders and half of those with dissociative disorders who experience problematic nightmares. Nightmares may be a side effect of medication in some cases, but there are likely other causal contributors too.

**Table 1:** Theorised routes between nightmares and psychiatric symptoms.

Theorised pathway	Theorised mechanistic account
Nightmares to psychiatric symptoms	Nightmares act as a stressor which triggers negative affective states.
	Catastrophic nightmare related appraisals (e.g. they are a sign of bad things that are going to happen) trigger daytime negative affect and associated unhelpful behavioural responses (e.g. avoidance and hypervigilance).
	Nightmares result in insomnia, which is causally related to a range of mental health problems.
Psychiatric symptoms to nightmares	Psychiatric symptoms place greater burden on the REM sleep system, which functions to consolidate emotional memories and experiences. A dysfunction in this system results in nightmares.
	Detachment type dissociation provides short term escape from distress. Yet this prevents the opportunity for emotional processing prior to sleep. The neural underpinnings for this emotional ‘braking system’ are deactivated during REM sleep, and hence an emotionally intense nightmare is more likely to ensue.
Nightmares and psychiatric symptoms are causally unrelated	Nightmares are symptom of PTSD only.
	Nightmares are a side effect of psychiatric medication.
	Nightmares and psychiatric symptoms are both a consequence of stressful life events

It is possible that both nightmares and psychiatric symptoms are caused by stress resulting from negative life events. Surprisingly, few studies have tested this hypothesis. In the month that followed the 1989 San Francisco Earthquake, students exposed to the earthquake reported significantly more nightmares than those who were not (Wood, Bootzin, Rosenhan, Nolen-Hoeksema, & Jourden, 1992). It is likely that a subgroup of these participants would have subsequently developed PTSD, but this was not assessed. In a separate study, retrospective ratings of the previous day's stress severity predicted nightmare occurrence and severity later that night (odds ratio = 1.11). However experiencing a nightmare also elevated stress severity ratings the following day (Garcia et al., 2021). It is possible that difficult and traumatic life events act as a trigger for both nightmares and psychiatric symptoms but that stress and nightmares subsequently develop a bidirectional maintenance cycle. Lastly, it remains possible that nightmares are not causally related to a range of psychiatric symptoms, but are simply a consequence of PTSD as suggested by diagnostic classification systems.

### 1.4 Review aims

The aim in the current review is to assess whether there is evidence for a causal relationship between nightmares and a range of psychiatric conditions. Currently the common clinical assumption is that nightmares are a consequence of waking distress, and hence the priority is to treat the mental health complaint. This review will consider whether this assumption is supported by research. The alternative direction of effect, from nightmares to psychiatric symptoms will be also assessed. Key symptoms of the main adult mental health disorders will be assessed including: PTSD, depression, anxiety disorders, schizophrenia spectrum disorders, bipolar affective disorder, dissociative disorders, personality disorders, and eating disorders. Suicidal behaviour disorder was also included owing to the well documented associations between nightmares and suicide. Symptoms of disorders typically exist on a spectrum of severity in the general population, including psychotic (Freeman et al., 2011; Johns et al., 2004) and dissociative experiences (Černis, Evans, Ehlers, & Freeman, 2021), which provides the opportunity to learn from studies of subclinical presentations in general population datasets. The repeated occurrence of nightmares which cause significant distress or impairment in functioning are diagnosable as nightmare disorder (American Psychiatric Association, 2013). However research rarely assesses diagnostic criteria, nor uses it as an inclusion criteria. Instead the spectrum of severity of nightmares is assessed commonly via assessment of nightmare frequency, nightmare distress or single items from PTSD assessments.

Two tiers of evidence are reviewed in considering causal associations between nightmares and psychiatric symptoms. The most convincing evidence for a causal relationship comes from intervening on the causal variable of interest and assessing the impact on the outcome (an interventionist-causal model approach; Kendler & Campbell, 2009). Hence randomised controlled experiments or trials that manipulate nightmares, or psychiatric symptoms, and measure the impact on the other are taken as the highest grade of evidence for a causal relationship in this review. Nightmares, for example, can be successfully treated using psychotherapy and the impact of psychiatric symptoms assessed (Casement & Swanson, 2012; Hansen, Höfling, Kröner-Borowik, Stangier, & Steil, 2013). Whilst inferring causation from longitudinal designs is more problematic, these studies are reviewed for the opportunity to detect novel associations that can be tested in future interventionist-causal tests.

The review will evaluate evidence that looks at both directions of the potential connection between nightmares and psychiatric symptoms. The key questions addressed are:

1. When nightmares are manipulated – in experimental studies or clinical trials - does that lead to changes in psychiatric symptoms (and vice versa)?
2. In longitudinal studies, does the occurrence or severity of nightmares predict the later occurrence of psychiatric symptoms (and vice versa)?

## 2 Method

Ovid Medline, Embase and PsycINFO were searched for English language papers published in peer-reviewed journals from 1980 onwards. A search was carried out on 20th August 2019 and an updated search on 21st September 2022 combining the search term nightmare* individually with each of the following key symptoms of key DSM-5 psychiatric disorders: depression (depress*), anxiety disorders (panic OR OCD OR anxiety OR phobia OR PTSD), dissociation (dissociat* OR multiple personality OR depersonali$ation OR dereali$ation), schizophrenia (delusion* OR hallucinat* OR psychosis OR psychotic OR schizophren*), suicide (suicid*) bipolar affective disorder (manic OR mania OR bipolar), eating disorders (eating disorders OR anorexia nervosa OR bulimia nervosa OR binge eating) and personality disorders (personality disorder). Duplicates were removed, following which the individual searches were combined. The database was then de-duplicated a second time to remove articles which had been detected in multiple searches.

Titles and abstracts were screened for the inclusion and exclusion criteria, and when needed the full text was read. Papers were required to include i) a quantitative measure of nightmares; ii) a quantitative measure of a psychiatric symptom; iii) an assessment of the relationship between nightmares and a psychiatric symptom; iv) a longitudinal design or experimental manipulation design; v) for manipulation studies only, a control group was required; as well as vi) a significant between group manipulation effect on either nightmares or psychiatric symptoms.

Exclusion criteria were i) literature reviews; ii) theoretical manuscripts; iii) single case reports; iv) qualitative studies; v) conference abstracts; vi) participants who experience nightmares and have an organic syndrome (i.e., learning disability, the dementias, brain injury) because these conditions may act as a confound in assessing the relationship between nightmares and psychiatric symptoms; and viii) a publication date prior to 1980.

We appraised the quality of studies using a custom-made set of appraisal questions designed to meet the needs of this review. Each paper was appraised for the quality with which it answered the review question based on whether i) the study was designed with the primary aim to assess the association between nightmares and a psychiatric symptom(s); ii) the inclusion criteria were appropriate for answering the review question (for example, whether those with severe psychiatric symptoms were excluded); iii) the study was powered to assess the relationship between nightmares and psychiatric symptoms; iv) validated measures of psychiatric symptoms and nightmares were used (i.e. a measurement tool for which the psychometric properties are published in a peer reviewed journal); and v) in longitudinal studies, whether the statistical analysis controlled for the baseline level of the outcome variable. This appraisal method was chosen over a pre-existing tool because many of the weaknesses of the studies derived from not being designed to answer the questions of this review. The study appraisal informed the structure of the results section. Better quality research for assessing the relationship between nightmares and psychiatric symptoms is highlighted, and reported earlier in the results section for each disorder. The quality of the evidence is considered alongside statistical tests of the relationship between nightmares and psychiatric symptoms.

## 3 Results

**Fig. 1:**
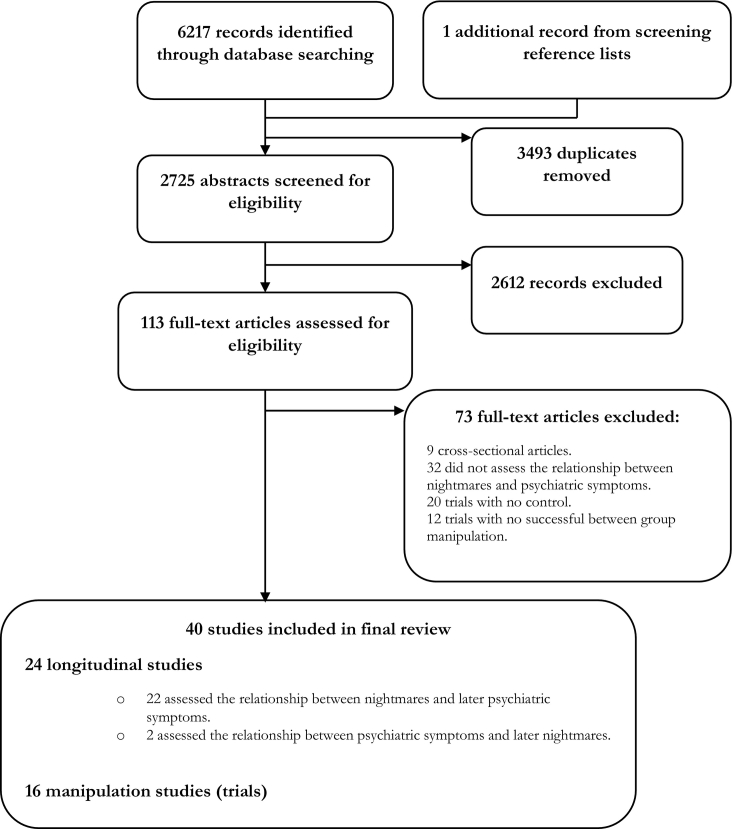
PRISMA flow diagram.

The search resulted in 6217 hits in addition to one additional article identified from a reference list. 2725 abstracts remained after removing duplicates. Over 2000 were excluded at abstract screening. Common reasons included cross-sectional design, single case descriptions, and assessment of nightmares in the context of an organic syndrome. After screening, 40 eligible papers remained (see Fig. 1 for PRISMA flow diagram). Twenty-four studies were longitudinal, of which 22 assessed the relationship between nightmares and later psychiatric symptoms and two assessed the reverse relationship.

All of the nightmare manipulation studies were treatment trials (Table 2). Thirteen out of the 16 trials treated nightmares and assessed the impact on psychiatric symptoms. PTSD (*n* = 10) and depression (*n* = 10) were the most common outcomes assessed followed by anxiety (*n* = 5), dissociation (*n* = 2), suicidal ideation (n = 2) and psychotic symptoms (n = 1). All trials were conducted in adult participants. No experimental studies were identified. None of the clinical trials were designed primarily for the purpose of assessing the impact on psychiatric symptoms. A methodological strength was the use of validated assessments of psychiatric symptoms, whereas a methodological weakness was that only half of the studies used a validated measure of nightmares. Three trials treated psychiatric symptoms (all were PTSD treatment trials) and assessed trauma-related nightmares using individual items from PTSD scales (Table 4).

**Table 2:** Controlled trials treating nightmares and assessing the impact on psychiatric symptoms.

Citation	Design	N	Sample	Assessments	Measure of nightmares	Measure of psychiatric symptoms	Key result for psychiatric symptoms	Change in psychiatric symptom?					
								Depr.	Anx.	PTSD	Dissoc.	Suic.	Psychosis
Ahmadpanah et al. (2014)	RCT testing: prazosin vs. hydroxyzine vs placebo.	100	Patients with PTSD and severe sleep disorders	Baseline and at 8 weeks (end of study)	PSQI item assessing bad dreams	PTSD (M.I.N·I)	Significant reductions in PTSD.	N/A	N/A	Yes	N/A	N/A	N/A
Burgess et al. (1998)	RCT testing: self-help exposure vs. self-help relaxation vs. waitlist control.	170	Nightmare sufferers	Baseline, 1-month and 6-month follow-up.	4-week nightmare diary (frequency and intensity rated from 0 to 8).	Phobia (fear questionnaire), depression (BDI).	Significant reductions in phobia and depression.	Yes	Yes	N/A	N/A	N/A	N/A
Davis and Wright (2007)	RCT testing: ERRT vs. waitlist control.	43	Individuals experiencing a traumatic event and nightmares	Baseline, 3 and 6-month follow-up	Trauma Related Nightmare Survey	PTSD (Structured Clinical Interview for DSM-IV: PTSD & Modified PTSD Symptom Scale Self Report), Depression (BDI).	Greater improvements in PTSD symptoms (*d* = 0.53) and depression (*d* = 0.59) following treatment versus waitlist control.	Yes	N/A	Yes	N/A	N/A	N/A
Davis et al. (2011)	RCT testing ERRT vs. waitlist control.	47	Trauma-exposed individuals	Baseline, 1-week post-treatment and 3 and 6-months.	Trauma Related Nightmare Survey.	PTSD (CAPS), Trauma related depression, anger/irritability and dissociation (Trauma symptom inventory).	Significant improvements in depression (*d* = 0.37) and PTSD (*d* = 0.39). No significant reduction in dissociation (*d* = 0.11, *p* < .05).	Yes	N/A	Yes	No	N/A	N/A
Krakow et al. (2000)	RCT testing IRT vs. waitlist control.	169	Female sexual assault survivors with PTSD and chronic nightmares.	Baseline and 3-month follow-up.	Nightmare Frequency Questionnaire (NFQ), Nightmare Effects Survey (NES).	PTSD (PSS).	The IRT group had significantly lower PTSD severity than control at 3 months. Within subject effect sizes: IRT *d* = 1.20, waitlist *d* *=* *0*.28).	N/A	N/A	Yes	N/A	N/A	N/A
Lancee et al. (2010)	RCT testing IRT vs. IE vs. recording (diary) vs. waitlist control	399	Those experiencing at least one nightmare or bad dream per week	Baseline and 11 weeks follow-up.	SLEEP-50	Anxiety (STAI) Depression (CES—D), PTSD (IES).	Nightmares treated with IRT led to significant improvements in anxiety (*d* = 0.25) compared to waitlist control, but not depression (*d* = 0.26) or PTSD (*d* = 0.11). Nightmares treated with IE led to significant reductions in depression (*d* = 0.56) but not anxiety (*d* = 0.13) or PTSD (*d* = 0.09) compared with wait list.	Mixed	Mixed	No	N/A	N/A	N/A
Lancee, Effting, & Kunze, 2021	RCT testing guided self-help imagery rehearsal therapy versus wait-list control.	70	Nightmare disorder according to DSM-5.	Baseline and post-treatment (5 weeks). IRT group followed up at 3 and 6 months.	Nightmare frequency questionnaire (NFQ), Nightmare distress and impact questionnaire (NDIQ).	Depression (PHQ-9), Anxiety (HADS-A).	No significant Treatment × Time interactions were found for depressive symptoms (PHQ; *F* = 1.68, *p* = .199, *d* = 0.30) or anxiety symptoms (HADS-A; *F* = 0.49, *p* = .488, *d* = 0.19).	Trend	No	N/A	N/A	N/A	N/A
Pruiksma et al. (2020)	Pilot RCT comparing ERRT with minimal contact control (MCC).	40	Active duty military personnel with nightmares	Baseline, end of treatment (5 weeks) and 1 month follow up	Trauma related nightmare survey	PTSD (PTSD checklist for DSM-5), depression (PHQ-9), suicide (depressive symptom index-suicide subscale)	ERRT led to medium effect size improvements in depression (*d* = −0.51) and marginal improvements in PTSD (*d* = −0.12) compared with MCC at post-treatment. MCC led to marginally greater changes in suicidal ideation than ERRT (*d* = 0.16). The study was not powered to detect statistically significant changes of this magnitude.	Yes	N/A	No	N/A	No	N/A
Raskind et al., 2013	RCT testing 8 weeks of prazosin vs. placebo control medication.	40 (34 analysed)	Veterans with chronic PTSD and severe trauma related nightmares.	Baseline, 4 and 8 week follow up. Only 8 week follow up yielded significant between group difference on nightmares.	Nightmare item of the CAPS PTSD scale.	PTSD (CAPS total), and depression (HAM—D) at baseline, 4 and 8 week follow up.	A trend towards reduction in depressive symptoms (*p* = .08). No significant reduction in PTSD at 8 weeks.	Trend	N/A	No	N/A	N/A	N/A
Raskind et al. (2013)	RCT testing 15 weeks of prazosin vs. placebo medication control.	67	Military personnel with PTSD and nightmares at least twice per week.	Baseline and 15 weeks.	Nightmare item of CAPS	PTSD (CAPS total) and depression (HAM-D and PHQ-9).	Significant between group effect on PTSD favouring the prazosin group.	Trend	N/A	Yes	N/A	N/A	N/A
Sheaves et al. (2019)	RCT comparing CBT for nightmares with treatment as usual (TAU).	24	Patients with weekly distressing nightmares and persecutory delusions and diagnosis of non-affective psychosis	Assessment at baseline and weeks 4 (end of treatment) and 8 (follow-up).	Nightmare severity (DDNSI, The Oxford Nightmare Severity Scale, daily nightmare logs).	Paranoia (GPTS), hallucinations (CAPS), negative affect (DASS-21), suicidal ideation (BSS), dissociation, (DES—B).	Medium effect size reduction in paranoia (*d* = −0.6), no effect on hallucinations (*d* = 0.1), reduction in dissociation (*d* = −0.8), mixed effects on negative affect. Suicidal ideation remained stable in CBT group but improved in TAU group (*d* = 0.3 at post-treatment, *d* = 0.7 at follow-up).	No	Mixed	N/A	Yes	No	Yes for paranoia, no for hallucinations
Taylor et al. (2008)	Within subjects crossover RCT testing Prazosin vs. placebo control	13	Outpatients with PTSD and nightmares.	Assessment and post 3 week treatment for each condition (separated by 1 week wash-out).	CAPS	PTSD Checklist-Civilian	Significant reduction in PTSD symptoms (*d* = 0.79).	N/A	N/A	Yes	N/A	N/A	N/A
van Schagen et al. (2015)	RCT comparing IRT plus TAU vs. TAU alone.	90	Patients with nightmares and a moderate to severe psychiatric disorder	Baseline, after IRT and 3-month follow-up.	Daily nightmare logs, the NFQ, Nightmare Distress Questionnaire and Nightmare Effects Survey.	General psychopathology (Symptom Checklist-90) and PTSD (Self-inventory list of PTSD symptoms)	Significant reduction in PTSD (*d* = 0.69) maintained at follow-up. Significant reduction in anxiety (*d* = 0.58), and depression (*d* = 0.55) at post-treatment, but fell short of significance at follow up. No significant reduction in agoraphobia.	Yes	Yes	Yes	N/A	N/A	N/A

Anx. = anxiety. Depr = depression. Dissoc = Dissociation. Suic = Suicidal ideation / behaviour. ERRT = exposure, relaxation and rescripting therapy. IE = imaginal exposure. IRT = imagery rehearsal training. PTSD = post-traumatic stress disorder. TAU = treatment as usual. RCT = randomised controlled trial. WL = waitlist control.

Of the longitudinal studies assessing the relationship between nightmares and later psychiatric symptoms suicidal ideation was the most common outcome assessed (*n* = 12), followed by depression (*n* = 8), PTSD (*n* = 6), psychotic experiences (n = 2) and borderline personality disorder (n = 1) (Table 3). Seven of the longitudinal studies assessing the path from nightmares to psychiatric symptoms included children and adolescents. Several of the studies had significant methodological shortcomings, in particular, only three studies used a validated assessment of nightmares, and around half did not control for baseline levels of the reported outcome. Methodological strengths of the longitudinal studies include the majority of studies having appropriate participant selection criteria for answering the review question and being purposefully designed to assess the relationship between nightmares and a psychiatric symptom.

**Table 3:** The longitudinal association between nightmares and psychiatric symptoms.

Citation	Design	N	Sample	Measure of nightmares	Measure of psychiatric symptoms	Main findings	Depr.	PTSD	Suicidal ideation / behaviour	Psychotic experiences
Bernert et al. (2017)	A 21-day assessment period with three time periods (baseline, 7 and 21 days).	50	Students aged 18 and older with a suicide attempt history and recent suicidal ideation.	Nightmare severity assessed by the DDNSI.	Suicide (Beck scale for suicide ideation).	Nightmares associated with higher severity of suicidal ideation at 7 and 21 day follow up, after controlling for depression and baseline suicidal ideation. Insomnia and nightmares combined accounted for two thirds of the variance in suicidal ideation (R^2^ = 0.69 at 7 days and 0.59 at 21 days). Nightmares were not a significant predictor of later mood variability.	N/A	N/A	Yes	N/A
Fisher et al. (2014)	Birth cohort study. Children's nightmares were assessed between ages 2.5 and 9 years. Psychotic experiences.	6796	Children and their mothers.	Maternal report (“In the past year, has your child regularly had nightmares?”). Aged 12, children were asked “Since your 12th birthday have you had any dreams that woke you up? Were they frightening?”.	Key psychotic experiences including hallucinations, delusions, thought interference (the psychosis-like symptom interview).	Frequent childhood nightmares predicted later psychotic experience at 12 years, after controlling for sex, family adversity, emotional or behavioural problems, IQ and potential neurological problems (OR = 1.16, *p* < .05).	N/A	N/A	N/A	Yes
Gerhart et al. (2014)	Assessed at baseline and 6 months later.	779	Palestinian adults (amid ongoing violent political turmoil)	1-Item of the PSS assessed trauma related nightmares.	PTSD (PSS), depression (PHQ-9).	Trauma related nightmares predicted later PTSD (β = 0.10, p < .05) and depression (β = 0.10, p < .05) but the reverse relationships were not significant.	Yes	Yes	N/A	N/A
Greene, Gregory, Fone, & White (2015)	Assessed at 5 years of age (parent report) and assessed for risk for depression at 34 years.	7437	Children and their parents.	Parental reports on the frequency of nightmares.	Self-reported depression (yes/no) and if yes, whether this was within the past year.	Parent reports of childhood nightmares at age 5 were not associated with depression over the past year at age 34 (OR: 1.03, 95% CI: 0.84–1.56).	No	N/A	N/A	N/A
Hedström et al. (2020)	Baseline and on average 16 years later	40,902 (69 suicides within the sample)	Swedish national march cohort	Nightmare frequency (dichotomous: never or seldom versus sometimes to always)	Depression (diagnosis from patient register), depressive symptoms (self reported feeling sad, low-spirited or depressed). Suicide (death register, 69 suicides registered within the sample).	Often or always having nightmares was associated with a significantly increased incidence of suicide. However, after adjustment (for seven covariates) statistical significance was lost. Among participants without a diagnosis of depression at baseline, the odds of depression during follow-up was higher among those who suffered from nightmares than among those who did not (OR 1.35, 95% CI 1.19–1.53).	Yes	N/A	Yes	N/A
Kobayashi & Delahanty (2013)	Sleep assessed two weeks after a trauma and PTSD at seven weeks	45 split by gender (female = 17, male = 28).	Participants admitted to a trauma centre	Nightmares assessed by the PSQI-addendum for PTSD	PTSD (CAPS)	Nightmares were associated with later PTSD in men (*r* = 0.53, *p* < .01) but the relationship was not significant in women (*r* = 0.30, *p* > .05).	N/A	Mixed	N/A	N/A
Lereya et al. (2017)	Sleep assessed when children were 2.5, 3.5, 4.8 and 6.8 years of age. Borderline personality disorder symptoms assessed age 11–12.	5544	Adolescents	Mother questionnaire report of nightmares (“In the past year has your child regularly had nightmares?” yes any sleep problem versus no sleep problem)	Borderline personality disorder (UK childhood interview for DSM-IV borderline personality disorder)	Having persistent childhood nightmares was associated with later borderline personality disorder symptoms in adolescence after controlling for sleep onset and maintenance problems and confounders including psychiatric diagnosis, emotional and behavioural problems, abuse and family adversity (adjusted OR = 1.62; 95% CI = 1.12–2.32).	N/A	N/A	N/A	N/A
Li et al. (2010)	Outpatients completed a comprehensive sleep assessment (alongside their clinical assessment). Medical notes were subsequently reviewed one year later.	1231	Psychiatric outpatients aged 18–65 from Hong Kong	Nightmare frequency assessed over the past year, determined on a 5-point scale (0 = none, to 4 = >3 times per week).	A suicide attempt was defined as an act of intentional self-harm to end one's life, as documented by case notes.	Recurrent nightmares predicted later suicide attempts (OR = 8.17, 95% CI, 1.06–63.13). The addition of comorbid insomnia to nightmares increased the one-year incidence of suicide risk (OR = 17.08; 95% CI, 2.64–110.40).	N/A	N/A	Yes	N/A
Li et al. (2012)	4-year naturalistic follow-up. Analysis controlled for age, marital status, psychiatric comorbidity, and severity of depressive and anxiety symptoms.	371	Remitted depressed outpatients (mean age 44.6 ± 10.4 years, female 81.8%)	Sleep questionnaire Nightmare Distress Questionnaire	Depression and anxiety (MINI at baseline, HADS at follow up), suicidality (MINI suicidality module).	Participants experiencing at least one nightmare per week at baseline were less likely to be remitted from depression at follow-up compared with those without nightmares (with nightmares: 29.8% versus without: 47.3%, *p* < .01).	Yes	N/A	Yes	N/A
Li et al. (2016)	Baseline and approximately one year later.	388	Outpatients diagnosed with schizophrenia spectrum disorders.	Frequent nightmares were defined as having nightmares of at least once per week over the past year	Suicide attempt recorded in medical notes. A suicide attempt was defined as a deliberate act of self-harm with the intention to end one's life.	Nightmare complaint alone did not predict the occurrence of suicide attempts, but the comorbidity of nightmares and insomnia was associated with the risk of suicide attempt over follow-up (adjusted HR = 11.10, p < .05).	N/A	N/A	No	N/A
Liu et al. (2019)	Baseline and 1 year follow up	7072	Adolescents	One survey question: in the past year how often did you have nightmares? Response option was 7 point likert scale from never to almost every night.	Silverman et al. (2007) suicidal behaviour measure.	Frequent nightmares at baseline were significantly associated with suicide attempts and non-suicidal self-injury one year later. After adjusting for demographics, depression, impulsivity and prior suicide attempt, the association remained significant for suicide attempts (OR = 1.96, 95% CI = 1.15–3.33) and non-suicidal self-injury (OR = 1.1.52, 95% CI = 1.10–2.08). Adjustment for insomnia and sleep duration yielded almost no change.	N/A	N/A	Yes	N/A
Liu et al. (2020)	Baseline and 1 year follow up.	6923	Adolescents	One survey question: “During the past month, how many times did you have nightmares?” The participant entered number of nights with nightmares.	Suicidal thoughts: “Have you ever seriously thought about suicide or killing yourself over the past 12 months?” (yes/no). Suicidal plans:”“Have you ever had a specific plan for how you would kill yourself over the past 12 months?” (yes/no), suicide attempt: “Have you ever tried to kill yourself over the past 12 months?” (yes/no). Depression (CES—D).	This is the same sample as Liu et al. (2019). The log odds of endorsing suicidal thoughts, plans or attempts at follow-up were all significantly higher in those endorsing frequent nightmares at baseline. The relationship was partially mediated by depression. Adjusting for covariates and baseline suicidal behaviour reduced the size of effects. The same set of analysis assessing the relationship between nightmares distress and suicidal behaviour yielded mixed results.	Yes	N/A	Yes	N/A
Neylan et al. (2020)	Baseline (in emergency department) and 2 and 8 weeks follow up.	666	Adults experiencing a motor vehicle collision	Two modified questions from the CAPS for DSM-IV assessing frequency and distress caused by unpleasant dreams. Questions asked about bad dreams in general in the 30 days leading up to the accident, rather than linked to a specific trauma.	PTSD and acute stress disorder (PTSD Checklist for DSM-5; PCL-5), Peritraumatic distress and dissociation (8-item short-form of the 13-item Peritraumatic Distress Inventory and the 5-item revised Michigan Critical Events Perception Scale), depression (PROMIS short form).	Nightmares were a significant predictor of 2 week acute stress disorder (ASD) and 8 week PTSD, whilst controlling for retrospective reports of pre-trauma PTSD and peritraumatic symptoms (2 week ASD OR = 1.3, 95% CI = 1.0–1.5, 8 week PTSD OR = 1.3, 95% CI = 1.1–1.6). Nightmares predicted major depressive episode at 8 weeks (OR = 1.3, 95% CI = 1.0–1.6), whilst controlling for pre-trauma PTSD and depression. The association held whilst controlling for peritraumatic symptoms (OR = 1.2, 95% CI = 1.0–1.5). Nightmares were not significantly associated with depression at two weeks post-trauma (OR = 1.2, 95% CI = 1.0–1.5).	Yes	Yes	N/A	N/A
Pigeon, Campbell, Possemato, & Ouimette (2013)	Assessed at baseline and 6 months following baseline.	80	Combat veterans with hazardous alcohol use and at least subthreshold PTSD.	1-Item of PTSD Checklist – Military Version Coded as positive if “moderately bothered” by trauma related nightmares.	PTSD (The PTSD checklist), depression (CES—D).	Bothersome trauma related nightmares predict later PTSD severity. Of those endorsing nightmares at baseline, 41% developed PTSD, compared with 10% of those without. Trauma related nightmares were not associated with later depression.	No	Yes	N/A	N/A
Sandman et al. (2017)	Finnish National FINRISK Study assessed between 1972 and 2012 and followed up in 2014 or at point of death.	71,068	i) General population of Finland and ii) war veterans.	Survey question asks: “During the past 30 days have you had nightmares?”, (“often”, “sometimes” or “never.”).	Cases of suicide were measured using the Causes of Death Registry of Finland.	The unadjusted hazard ratio of suicide for persons who reported frequent nightmares compared with those who did not was 2.63 (*p* < .001), after adjustments this reduced to 1.84 (*p* = .010). The presence or absence of war veterans did not affect the association.	N/A	N/A	Yes	N/A
Shi et al. (2021)	Baseline and 6 months later	11,740	College students	Survey question: How often did you have nightmares during the past month (1 = never, 4 = more than three per week).	Survey questions: “Have you ever seriously thought about suicide during the past 12 months”, “Have you ever tried to kill yourself during the past 12 months” (yes/no). Similar questions were used to assess suicidal behaviours over the past 6 months at follow-up.	Endorsing frequent nightmares was associated with later suicidal ideation (OR: 1.69) and attempts (OR: 2.40) after controlling for a range of covariates including depression and baseline measure of the outcome.	N/A	N/A	Yes	N/A
Sjöström et al. (2009)	Assessed nightmares at baseline and two months follow up. Repeat suicide attempts assessed at 2 years follow-up.	165	Psychiatric inpatients admitted for suicide attempt aged between 18 and 69 years.	1-item of the Uppsala Sleep Inventory answered on a five-point frequency scale (1 = never, to 5 = very often). Score ≥ 4 at both baseline and follow-up was considered a persistent sleep disturbance.	Suicide attempt data taken from medical records. Psychiatric symptoms (SCID for DSM-IV and the Comprehensive Psychopathological Self-Rating Scale for Affective Syndromes).	Having frequent nightmares at baseline predicted repeat suicide attempts (OR = 3.15, 95% CI, 1.51–6.57). Persistent nightmares further heightened the risk (OR = 5.20, 95% CI, 1.91–14.13). Results remain even after adjusting for sex, axis-I diagnosis, depression, anxiety, PTSD and anti-depressant drugs. Decrease in odds ratio (OR = 2.28) after controlling for depression.	N/A	N/A	Yes	N/A
Tanskanen et al. (2001)	Finnish National FINRISK Study assessed between 1972 and 1992 and followed until December 31, 1995, or death	36,211	General population of Finland.	Survey question asks: ““How often have you had nightmares during the past month?” (“frequently,” “occasionally,” or “not at all.”).	Cases of suicide were measured using the Causes of Death Registry of Finland	Significant association between nightmares and later death by suicide. Among subjects having nightmares occasionally the adjusted relative risk of suicide was 57% higher, and among those reporting frequent nightmares 105% higher compared with subjects reporting no nightmares at all.	N/A	N/A	Yes	N/A
Thompson et al. (2015)	Follow up from Fisher et al. (2014). Children were interviewed at ages 12 and 18 years about nightmares and psychotic experiences.	4720	Children and their mothers.	Postal questionnaires completed by mothers when children aged 2.5–9 (“In the past year, has your child regularly had nightmares?”). At aged 12 children were asked “Since your 12th birthday have you had any dreams that woke you up? Were they frightening?”.	Key psychotic experiences including hallucinations, delusions, thought interference (the psychosis-like symptom interview).	Nightmares at 12 was associated with psychotic experiences age 18 when adjusting for IQ, family adversity, psychiatric disorders, depression, child abuse enuresis and psychotic experiences at 12 (OR = 1.62, 95% CI 1.19–2.20). The effect was larger for persistent psychotic experiences (at age 12 and 18 years, OR: 3.87 95% CI: 2.30–6.51).	N/A	N/A	N/A	Yes
van Liempt et al. (2013)	Assessed prior to military deployment to Afghanistan and at 6 months post-deployment.	453	Dutch service members (military)	1-Item of the Self-Rating Inventory for PTSD (“I had bad dreams”).	PTSD (SRIP).	Pre-deployment nightmares predicted later PTSD symptoms at six months post-deployment (OR = 2.99, 95% CI: 1.10–8.55), after controlling for pre-deployment PTSD, early trauma, mood and anxiety.	N/A	Yes	N/A	N/A
Wittmann et al. (2010)	Assessed at 10 days, 2 months, and 6 months after a traffic accident.	32	Children who experienced traffic accidents (Age 11.9; SD = 2.4; range = 7–15 years)	Trauma nightmare frequency scale of Clinician-Administered PTSD Scale, child and adolescent version, 1 item.	PTSD (CAPS child and adolescent version). Depression (children's depression inventory).	Trauma related nightmares ten days post-accident predict later PTSD total score two months post-accident, but not depression.	No	Yes	N/A	N/A
Wong et al. (2011)	Sleep problems assessed age 12–14. Suicidal thoughts and self-harm assessed age 15–17.	391	Children of parents who are alcoholics	One question on the Youth Self Report	Two items on the Youth Self Report.	Nightmares at age 12–14 did not predict later suicidal ideation or self-harm when ‘trouble sleeping’ was included in the model.	N/A	N/A	No	N/A

Anx. = anxiety. Depr = depression. PTSD = post-traumatic stress disorder.

**Table 4:** Controlled trials treating a psychiatric symptom and assessing the impact on nightmares.

								Change in nightmares following treatment of psychiatric symptom?
Citation	Design	N	Sample	Assessments	Measure of nightmares	Psychiatric symptom manipulated (measure)	Key findings	PTSD treatment
Gutner et al. (2013)	RCT comparing prolonged exposure (PE) and cognitive processing therapy (CPT)with minimally attention (MA) control.	171	Female rape victims with PTSD	Baseline, post-MA (6 weeks), post-treatment, 3-months, 9-months and LTFU	CAPS	CAPS	Large reductions in nightmares following treatment of PTSD using CPT or PE.	Yes
Silver et al. (1995)	Controlled trial testing PTSD treatment alone (control) vs. additional treatment with i) EMDR, ii) Biofeedback and iii) Relaxation training.	100	Inpatients with PTSD.	Baseline, at admission, 2 months later and at discharge 90 days after admission.	1-item of the Problem Report Form (PRF) rating severity of nightmares over past 7 days on 6 point likert scale.	PTSD flashbacks	Significant reduction in nightmares (*p* < .05).	Yes
Woodward et al. (2017)	RCT comparing cognitive therapy for PTSD with emotion-focused supportive therapy, and a waitlist.	121	Patients with PTSD.	Baseline and 14 weeks.	One item each from two PTSD scales (CAPS and PDS)	PTSD (PDS).	CT-PTSD led to significant large effect size reductions in nightmares when compared with emotion focused supportive therapy and waitlist control (partial η2 = 0.15–0.16).	Yes

EMDR = Eye movement desensitization and reprocessing. PTSD = post-traumatic stress disorder.

**Table 5:** The longitudinal association between psychiatric symptoms and nightmares.

Citation	Assessment schedule	N	Sample	Measure of psychiatric symptom	Measure of nightmares	Key findings	Depr.	Anx.	PTSD	Psychosis
Levin and Fireman (2002)	Baseline assessment of psychiatric symptom and nightmare log over subsequent 3 weeks.	116	Students aged 18 or older.	Depression (BDI), anxiety (STAI), dissociation (DES), schizotypy (Perceptual Aberration-Magical Ideation scale, PerMag) and OCD, depression, anxiety, phobia, paranoia (SCL-90R).	Nightmare frequency (nightmare log) and distress (Nightmare distress Scale).	Individuals who reported ≥3 nightmares (frequent nightmares) across the 3 weeks had significantly higher baseline scores on OCD, paranoia, psychoticism, dissociation and schizotypy. Depression and anxiety were not significantly higher at baseline in those reporting frequent nightmares.	No	Mixed	N/A	Yes
Short, Allan, Stentz, Portero, & Schmidt, 2018	Baseline and ecological momentary assessment over 8 days.	30	Adults with PTSD, 93% had other psychiatric co-morbidities.	PTSD (SCID and PTSD Checklist), anxiety (BAI), depression (BDI-II).	One item related to traumatic nightmares from the PSQI PTSD addendum.	Daytime PTSD symptoms predicted nightmares later that night after controlling for baseline PTSD. Baseline depression, daily anxiety and daily stressors did not predict subsequent nightmares.	No	No	Yes	N/A

Anx. = anxiety. Depr = depression. PTSD = post-traumatic stress disorder.

Two studies assessed the longitudinal relationship between psychiatric symptoms and later nightmares (Table 5). Within these two studies depression (n = 2), anxiety (n = 2), PTSD (n = 1) and psychotic experiences (n = 1) were assessed. Of these, Short, Allan, Stentz, Portero, & Schmidt, 2018 was methodologically stronger across the appraisal criteria and hence these results are given greater weight.

No longitudinal or manipulation studies investigated the path from psychiatric symptoms to nightmares in children and adolescents. In addition, the search detected no longitudinal or manipulation studies assessing the association between nightmares and panic disorder, bipolar disorder, eating disorders (in either direction) and the only investigation into nightmares and personality disorders was one longitudinal study focusing on borderline personality disorder specifically.

Many of the studies reported multiple psychiatric symptom outcomes and hence there is overlap in studies across different mental health conditions.

### 3.1 PTSD

#### 3.1.1 From nightmares to PTSD

The majority of empirical studies assessing PTSD investigated the consequences for PTSD symptoms of treating nightmares (*n* = 10). The studies that were stronger methodologically (e.g. Krakow et al., 2000; van Schagen, Lancee, De Groot, Spoormaker, & Van Den Bout, 2015) support the view that successfully treating nightmares alleviates other PTSD symptoms too. The methodologically strongest study for assessing the relationship between nightmares and PTSD was conducted by Krakow et al. (2000). A large sample of 167 participants with both nightmares and clinical levels of PTSD were randomly allocated to a seven-hour psychological intervention focused on nightmares, or waitlist control. In the intervention participants were requested to refrain from sharing a full disclosure of the traumatic event and only brief details relevant to nightmares were shared (e.g., being assaulted in the bedroom and hence keeping the lights on at night,), hence attempting to isolate nightmares from other PTSD symptoms. The treatment led to significant reductions in nightmare frequency, nightmare-related impairment, and large reductions in PTSD symptoms (*d* = 0.94) when compared with wait-list control. The results are supported by a second trial (van Schagen et al., 2015) which similarly tried to exclude exposure to the trauma memory. Ninety patients experiencing nightmares and a range of psychiatric disorders (17% had an anxiety disorder) took part. Compared with treatment as usual (TAU) the psychological treatment (lasting six hours) led to moderate reductions in PTSD (*d* = 0.69). A relative strength of the study by van Schagen et al. (2015) is the removal of the question relating to ‘bad dreams’ from the PTSD assessment, suggesting that the treatment of nightmares impacts on other PTSD symptoms.

One medication trial has the methodological benefit of a placebo control condition. Raskind et al. (2013) recruited 67 patients with PTSD and nightmares and tested the alpha1-adrenergic antagonist prazosin (originally developed to treat high blood pressure) against a placebo control. They reported a significant reduction in trauma-related nightmares and PTSD, favouring the prazosin group. The result remained significant after removal of the nightmare item from the PTSD assessment. However the distal method of action of prazosin is unclear. It may target PTSD more broadly, rather than isolating nightmares specifically.

The majority of longitudinal studies have tested the hypothesis that trauma-related nightmares are associated with the later severity of PTSD (*n* = 6). Just one study assessed the reverse relationship (Short, Allan, Stentz, Portero, & Schmidt, 2018). The strongest evidence comes from van Liempt, van Zuiden, Westenberg, Super, and Vermetten (2013) who assessed nightmares in military personnel (*N* = 453) prior to being deployed (i.e., before potential trauma) and report that endorsing the item “I have bad dreams” (on a PTSD assessment) was associated with almost three times greater odds of later post-deployment PTSD symptoms (odds ratio, OR = 2.99). The strengths of the study are the assessment of nightmares prior to the index trauma, the large participant group, and that pre-deployment PTSD, anxiety, mood symptoms and early trauma were all controlled for, hence mitigating the effects of key confounds. Their result is supported by Neylan et al. (2020) who recruited adults from an emergency department (*N* = 666) who had experienced a motor vehicle collision. General nightmares (as opposed to trauma related nightmares) in the month prior to the trauma predicted later acute stress disorder (two weeks post-trauma) and PTSD (at eight weeks) whilst controlling for pre-trauma PTSD and peritraumatic symptoms. These two studies suggest that nightmares prior to experiencing a traumatic event elevate the subsequent risk for developing PTSD.

A small study (*N* = 32) with children (Wittmann, Zehnder, Schredl, Jenni, & Landolt, 2010) showed that nightmares in the 10 days following trauma were associated with later PTSD symptoms 2 and 6 months later, whilst controlling for baseline acute stress disorder symptoms.

#### 3.1.2 From PTSD to nightmares

Three clinical trials reported a significant reduction in nightmare severity following successful treatment of PTSD (Gutner, Casement, Gilbert, & Resick, 2013; Silver, Brooks, & Obenchain, 1995; Woodward et al., 2017). Woodward et al. (2017) was methodologically strong owing to a large sample size (*N* = 121) and the fact that baseline severity of nightmares was controlled for. A significant large effect size reduction in nightmares is reported following up to 20  hours of psychological treatment for PTSD in which sleep was not directly targeted, when compared with emotion-focused supportive therapy and a wait-list control (partial η^2^ = 0.15–0.16, Woodward et al., 2017). This study supported earlier findings from Gutner et al. (2013) who recruited female participants with a more specific trauma (rape victims) with PTSD, and compared both prolonged exposure and cognitive processing therapy against a minimal attention control comparison; though the authors note that sleep disturbance did not reach the point of full remission following treatment for PTSD. All three trials used one question to assess nightmares rather than a validated scale. Gutner et al. (2013) and Silver et al. (1995) did not use intention-to-treat analysis, and Silver et al. (1995) was a service evaluation which therefore did not use randomisation. Lastly, it is unknown from the available evidence whether treating PTSD affects all nightmares or only those that are trauma related.

One longitudinal study assessed the direction from PTSD to nightmares:, Short, Allan, Stentz, Portero, & Schmidt, 2018 report that daytime PTSD symptoms predicted subsequent trauma related nightmares after controlling for baseline PTSD (but not nightmares specifically), using an ecological momentary assessment design.

#### 3.1.3 Summary

Taken together, the treatment of nightmares leads to moderate reductions in PTSD symptoms more broadly and there is evidence that pre-trauma nightmares might be an early risk factor for the later onset of PTSD after trauma exposure. It is of course possible that pre-trauma nightmares reflect a PTSD process which is disrupted and measurable even prior to trauma exposure, in the same way that negative core beliefs might leave one prone to the development of PTSD. In this way nightmares could reflect a general vulnerability to later PTSD rather than a specific causal process. No tests have assessed the impact of treating idiopathic nightmares prior to trauma exposure on the later development of PTSD. There are also fewer studies assessing the route from PTSD to nightmares, but initial evidence supports a bidirectional cycle between nightmares and PTSD.

### 3.2 Suicide

#### 3.2.1 From nightmares to suicide

There have been just two small pilot randomised controlled trials assessing suicidal ideation in nightmare treatment trials. One trial recruited a group of military personnel (*N* = 40, Pruiksma et al., 2020) and the second recruited patients experiencing psychosis (*N* = 24, Sheaves et al., 2019). In neither study were participants selected on the basis of suicidal ideation. In the Pruiksma et al. (2020) trial those with significant suicidal ideation were actually excluded. Hence both trials suffered floor effects for assessing change in suicidal ideation. However, the results across both pilot trials were consistent but contrary to hypotheses: suicidal ideation improved in the control group but remained relatively stable in the group who received treatment for nightmares. Whilst there is a clear caveat that these are small pilot trials and suffer floor effects, there is some indication that nightmares may be causally related to suicide, but in the opposite from the hypothesised direction - treating nightmares may actually prevent natural recovery from suicidal ideation.

The longitudinal studies are inconsistent with the results of the randomised controlled trials. There is substantial longitudinal evidence that nightmares predict the later occurrence of suicidal ideation (Bernert, Hom, Iwata, & Joiner, 2017; Li, Lam, Chan, Yu, & Wing, 2012; Liu, Yang, Liu, & Jia, 2020; Shi et al., 2021), attempts (Li et al., 2010; Liu et al., 2019; Liu et al., 2020; Shi et al., 2021; Sjöström, Hetta, & Waern, 2009), and completed suicides (Hedström et al., 2020; Sandman et al., 2017; Tanskanen et al., 2001). The quality of the longitudinal evidence is good. Participant groups were mostly large ranging from 50 (Bernert et al., 2017) to over 71,000 (Sandman et al., 2017). Several studies controlled for baseline levels of the suicide outcomes (Bernert et al., 2017; Liu et al., 2019; Liu et al., 2020; Shi et al., 2021) or measures of the severity of psychiatric symptoms more generally (e.g., depression) (Li, Lam, Chan, Yu and Wing, 2012, Li, Lam, Yu, Zhang and Wing, 2010; Sandman et al., 2017; Sjöström et al., 2009; Tanskanen et al., 2001). The association between nightmares and later suicide outcomes also held across the short term (one week; e.g., Bernert et al., 2017) and longer term (at least one year later; e.g., Sjöström et al., 2009; Li et al., 2010). It was reported across participant groups including adults endorsing baseline suicidal ideation (Bernert et al., 2017) or a recent attempt (Sjöström et al., 2009) as well as psychiatric patients (Li, Lam, Chan, Yu and Wing, 2012, Li, Lam, Yu, Zhang and Wing, 2010), large birth cohorts (Hedström et al., 2020; Tanskanen et al., 2001) and an adolescent group (Liu et al., 2019; Liu et al., 2020). The size of effects is moderate: in an inpatient group recently attempting suicide, frequent nightmares at baseline were associated with three times the odds of a repeat suicide attempt over the next two years (Sjöström et al., 2009), and recurrent nightmares over the past year in a psychiatric outpatient group was associated with eight times greater odds of later attempting suicide, though baseline suicidal ideation was not controlled for in this study (Li et al., 2010).

Evidence from both Sjöström et al. (2009) and Liu et al. (2020) indicate that negative affect is a partial mediator of the relationship between nightmares and suicidal ideation and attempts. Insomnia is another plausible candidate though findings are mixed. Liu et al. (2019) reported that adjusting for insomnia and sleep duration had no effect on the relationship. Whereas in Li et al. (2016) the relationship between nightmares and suicide attempts was only significant in those with comorbid insomnia.

Two studies did not find a significant direct relationship between baseline frequent nightmares and later suicidal attempts (Li et al., 2016; Wong, Brower, & Zucker, 2011). Li et al. (2016) recruited a large participant group with schizophrenia spectrum disorders (*N* = 388). Baseline nightmares were not associated with later suicide attempts (assessed by reviewing case notes). Wong et al. (2011) recruited a large group of adolescents (*N* = 391) aged 12–14 and found that nightmares did not predict suicidal ideation three years later. The prevalence of nightmares and suicidal ideation in the group were low and the findings are at odds with the findings from the larger study of Liu et al. (2019, 2020).

#### 3.2.2 From suicidal ideation to nightmares

No studies have assessed the direction from suicidal ideation, or attempted suicides to nightmares.

### 3.3 Depression

#### 3.3.1 From nightmares to depression

Ten trials assessed the impact on depression of treating nightmares. The findings are mixed but the better quality studies support the view that successfully treating nightmares results in moderate improvements in depressive symptoms (e.g. Davis & Wright, 2007; Pruiksma et al., 2020; van Schagen et al., 2015). No trial recruited participants on the basis of having depression, but in three trials the mean depression scores were in the moderate range at the start of treatment (Burgess, Gill, & Marks, 1998; Davis & Wright, 2007; Pruiksma et al., 2020). Two of the trials reported a significant effect of treating nightmares on depression (*d* = 0.59; Davis & Wright, 2007, Burgess et al., 1998) and the third was a pilot trial finding a moderate treatment effect (*d* = 0.51; Pruiksma et al., 2020). In all three of these trials the mean depression scores in the treatment group fell from the moderate to mild range following successful treatment of nightmares, but remained stable in the moderate range for the control group. Pruiksma et al. (2020) kept medication stable through the duration of the trial, suggesting that the improvements in nightmares and depression are unlikely due to changes in medication. Two further studies support the conclusion that treating nightmares may lessen depressive symptoms (Davis et al., 2011; van Schagen et al., 2015). In the van Schagen et al. (2015) trial just under a fifth of participants (total *N* = 86) had a primary diagnosis of a mood disorder. Imagery rehearsal (IR), a cognitive behavioural treatment technique specifically designed to treat nightmares, led to a moderate improvement in depression compared with TAU (*d* = 0.55). In two trials Lancee et al. (2010, 2020) report that exposure therapy (*d* = 0.56) and IR lead to reductions in depression (*d* = 0.26, *d* = 0.30) compared with waitlist controls, however the results for IR fell short of statistical significance.

Improvements in depression may not hold across all psychiatric populations: in a pilot trial with patients experiencing psychosis (where co-occurring depression is common, and depression scores fell into the moderate range at baseline), there was no clear effect on depressive symptoms of treating nightmares (Sheaves et al., 2019). It is possible that there are other more proximal contributors to depression in this group which are not targeted by the nightmares treatment, e.g., loss of social roles such as employment (Rooke & Birchwood, 1998) and a perceived lack of control over psychotic symptoms (Birchwood, Mason, Macmillan, & Healy, 1993).

Almost all of the longitudinal studies tested the hypothesis that baseline nightmares might be a risk factor for later depressive symptoms (*n* = 8). Findings were mixed and the quality of studies for addressing the question was poor overall, for example only one study used a validated psychometric assessment for nightmares. All but one study used a single question to assess nightmares. Li et al. (2012) conducted the most methodologically strong study: a validated measure of depression was used, baseline depression was controlled for, and a general (rather than trauma specific) nightmare measure was used. Participants had all experienced depression which had subsequently remitted (*N* = 371). The group who did not experience nightmares at baseline were more likely to remain remitted four years later than those experiencing weekly nightmares. Three other large cohort studies support the association between nightmares and later depression (Hedström et al., 2020; Liu et al., 2020; Neylan et al., 2020).

#### 3.3.2 From depression to nightmares

No studies have manipulated mood and assessed the impact on nightmares. Three studies provide preliminary evidence that there is no longitudinal relationship between depression and the later occurrence of subsequent nightmares (Gerhart, Hall, Russ, Canetti, & Hobfoll, 2014; Levin & Fireman, 2002; Short, Allan, Stentz, Portero, & Schmidt, 2018). However there is preliminary evidence that depression is moderately correlated with the degree of distress in later nightmares (Levin & Fireman, 2002). No trials treated depression and assessed the impact on nightmares.

### 3.4 Psychosis

#### 3.4.1 From nightmares to psychotic experiences

One pilot randomised controlled trial (*N* = 24) tested a treatment for nightmares in a patient group experiencing persecutory delusions (Sheaves et al., 2019). Successful treatment of nightmares (*d* = −1.06*,* 95% C·I = -12.6;-1.3) led to moderate effect size reductions in paranoia compared with TAU, though the wide confidence intervals merit caution (*d* = −0.6*,* 95% C·I = -43.2;1.7). No effect was found on hallucinations. Key medications were stable in both groups across the course of the trial, but the sample size of this pilot trial is small.

Two longitudinal studies followed the same birth cohort and provide good evidence that childhood nightmares are a risk factor for later psychotic experiences (Fisher et al., 2014; Thompson et al., 2015). Fisher et al. (2014) report a significant association with psychotic experiences at age 12, after adjusting for a range of potential confounding variables including affective symptoms and psychiatric disorders. Thompson et al. (2015) reported that the odds of reporting psychotic experiences at age 18 was 1.62 times higher in those reporting nightmares at age 12 than those who did not, whilst controlling for baseline psychotic experiences, child abuse and psychiatric disorders. However only very brief, unvalidated assessments of nightmares were used.

Given initial indications that nightmares are a measurable vulnerability factor for the development of later psychotic experiences, and particularly paranoia, further enquiry into the relationship is indicated. One issue not addressed by the current literature is the potential for shared causal factors which underpin both nightmares and psychotic symptoms. For example, there is evidence for shared genetic liability to both psychotic experiences and childhood nightmares (Reed et al., 2019).

#### 3.4.2 From psychotic experiences to nightmares

No studies were found which manipulated psychotic experiences and report the effect on nightmares. Levin and Fireman (2002) provide initial evidence that psychotic experiences predict subsequent nightmares, but baseline nightmare severity was not controlled for.

### 3.5 Other anxiety disorders

#### 3.5.1 From nightmares to other anxiety disorders

Five trials have successfully intervened on nightmares and assessed anxiety as an outcome. The better quality research provides evidence that treating nightmares results in small to moderate improvements in anxiety (e.g. Lancee et al., 2010; van Schagen et al., 2015). Lancee et al. (2010) conducted the largest trial (*N* = 399) using validated measures of both nightmares and anxiety. IR and exposure to nightmare content were compared with recording and wait list control. They reported small but significant reductions in anxiety following IR treated nightmares, compared with recording of nightmares and wait list control (Cohen's *d* = 0.31 and *d* = 0.25 respectively). The exposure intervention led to small but significant (*d* = 0.20) improvements in anxiety compared with a nightmare recording condition, but not when compared to the wait list control condition. van Schagen et al. (2015) recruited participants with psychiatric symptoms and results support the view that treating nightmares reduces anxiety when compared with TAU (*d* = 0.58). A small but non-significant effect was found on agoraphobia (*d* = 0.25), but the trial was not powered to detect effects of this size. Lancee, Effting, & Kunze, 2021 report a very small but non-significant improvement in anxiety from self-help IR treatment compared with wait-list control (*d* = 0.19), but the trial was not powered to detect effect sizes of this magnitude.

Burgess et al. (1998) assessed simple phobias and report a significant reduction following nightmare treatment compared with both relaxation and waitlist control conditions (*N* = 170). Lastly, in patients experiencing persecutory delusions Sheaves et al. (2019) report a small treatment effect of cognitive behavioural therapy (CBT) for nightmares on anxiety at the end of treatment (*d* = 0.45), but not at follow up, despite the treatment effect for nightmares being maintained.

#### 3.5.2 From other anxiety disorders to nightmares

Just one study has assessed anxiety as a risk factor for later nightmares, but the study did not control for baseline severity of nightmares. Levin and Fireman (2002) report that baseline SCL-90 assessed anxiety (but not STAI assessed anxiety) and obsessive compulsive symptoms were higher in participants subsequently reporting weekly nightmares compared with less frequent, or no nightmares. There was no association between simple phobia and nightmares.

### 3.6 Dissociation

#### 3.6.1 From nightmares to dissociation

Despite more than half of people with dissociative disorders experiencing problematic nightmares (Agargun et al., 2003), there is little research assessing the association between nightmares and dissociation. Two trials have assessed dissociation as an outcome when testing the psychological treatment of nightmares, but neither trial had dissociation as an eligibility criteria. Davis et al. (2011) report a very small but non-significant effect of a psychological nightmare treatment on dissociation (*d* = 0.11) in trauma exposed individuals, when compared with waitlist control. Sheaves et al. (2019) recruited patients (*N* = 24) with persecutory delusions, within which dissociative symptoms were commonly present. A large treatment effect on dissociation was found, albeit with wide confidence intervals (*d* = −0.84, 95% C.I. = −1.4;0.1).

#### 3.6.2 From dissociation to nightmares

No studies assessed the path from dissociation to nightmares.

### 3.7 Personality disorders

#### 3.7.1 From nightmares to personality disorders

Nightmares are a recognised problem for around half of patients with borderline personality disorder (BPD) (Semiz, Basoglu, Ebrinc, & Cetin, 2008). Yet only one study has investigated a potential causal relationship between nightmares and BPD symptoms. Lereya, Winsper, Tang, and Wolke (2017) found that persistent childhood nightmares (assessed at three time points) were associated with later borderline personality disorder symptoms in adolescents (adjusted OR = 1.62, 95% C.I. = 1.12–2.32). Whilst baseline BPD symptoms were not controlled for, a range of associated confounders were, including: psychiatric diagnosis, emotional and behavioural problems, abuse and family adversity. Strengths of the study include the use of a validated semi-structured interview to assess BPD symptoms, and the large sample size, whereas a relative weakness is the use of a single item to assess parent reported nightmares. There have been no controlled trials of treatments for nightmares for patients with BPD, and no other personality disorders have been investigated for their potential causal relationship with nightmares.

#### 3.7.2 From personality disorders to nightmares

No research has investigated the path from personality disorders to nightmares.

## 4 Discussion

*“People don't realise how much it can impact you by having nightmares all the time [pause] and how it affects your day [..]. You know that knock on effect”.*

‘Amy’ – an outpatient experiencing psychosis and nightmares

It is striking given the historical fascination with nightmares that there are so few studies that can inform understanding of their effects. Only sixteen small clinical trials have potential to inform the understanding of the possible causal relationship between nightmares and psychiatric symptoms. Almost all of the research has assessed the direction of effect from nightmares to other mental health difficulties, rather than the reverse direction of effect. Depression (*n* = 10 studies), PTSD (n = 10) and anxiety (*n* = 5) were the most commonly assessed psychiatric outcomes in nightmare treatment trials. However participant pools are biased towards PTSD: two thirds of nightmare treatment trials were conducted in participant groups who have either been exposed to a trauma, or have co-occurring PTSD, which limits the generalisability of results, particularly for depression. It is clear that nightmares are eminently treatable alongside co-occurring psychiatric symptoms, which is what almost all of the trials set out to assess. The subsequent effects on other psychiatric symptoms were almost never the primary goal of trials – hence this review is an opportunistic review of secondary outcomes. The trials are not powered for their effects on other psychiatric conditions. Overall the findings support the view that treating nightmares likely improves threat-based disorders, with moderate reductions observed in PTSD (van Schagen et al., 2015), small to moderate reductions in anxiety (Lancee et al., 2010; van Schagen et al., 2015) and there is preliminary evidence that treating nightmares leads to moderate reductions in paranoia (Sheaves et al., 2019). Despite the common clinical assumption that nightmares are a consequence of daytime distress, just three trials tested the pathway from psychiatric symptoms to nightmares. They provide preliminary evidence that there are large reductions in trauma-related nightmares after PTSD treatment (Gutner et al., 2013; Silver et al., 1995; Woodward et al., 2017), but the effect on nightmares of treatments for other psychiatric problems have not been tested.

It is understandable that PTSD was one of the most common psychiatric outcomes investigated, given that nightmares are a recognised symptom of the disorder. The results of this review suggest that treating one isolated symptom of PTSD - trauma-related nightmares - leads to moderate reductions in PTSD overall, and conversely, treating PTSD leads to large effect size improvements in trauma related nightmares. Whilst tempting to assume that the causal pathway is stronger from other PTSD symptoms to nightmares than the reverse, the comparison should be interpreted cautiously given that trauma focused CBT for PTSD has a greater effect on PTSD symptoms than CBT for nightmares does on nightmares (i.e., it is a stronger manipulation). Factor analytic studies of PTSD symptomatology confirm that trauma-related nightmares load onto a latent ‘intrusion’ factor (Wang et al., 2017) suggesting that there may be shared causation between nightmares and other intrusion symptoms. The fact that other PTSD symptoms improve following treatment of nightmares suggests either that trauma-related nightmares causally interact with other PTSD symptoms (as asserted by network models of psychopathology; Borsboom & Cramer, 2013), or that the nightmare treatment alters underlying mechanisms which give rise to both nightmares and other PTSD symptoms.

A question not addressed by the current review is whether nightmares which are not trauma related (‘idiopathic nightmares’) also share a bidirectional relationship with PTSD symptoms. No studies assessed whether successfully treating PTSD improves idiopathic nightmares, but there is preliminary evidence from an uncontrolled trial that they do not (Belleville, Guay, & Marchand, 2011). CBT for PTSD showed significant reductions in trauma-related nightmares (the mean severity score for trauma-related nightmares halved following treatment), but bad dreams unrelated to trauma were not significantly affected (Belleville et al., 2011). This finding requires testing in a controlled trial. A second key causal test which is missing from the evidence base is whether treating idiopathic nightmares prior to trauma reduces the risk of subsequently developing PTSD, particularly in groups where exposure to trauma is high (e.g., military personnel). There are plausible reasons to think that treating idiopathic nightmares might causally impact on PTSD: first, experimental studies have demonstrated that the function of REM sleep is for fear extinction learning (Menz, Rihm, & Büchel, 2016), second, fragmented REM sleep in the aftermath of trauma predicts the later development of PTSD (Mellman, Bustamante, Fins, Pigeon, & Nolan, 2002) and third, a longitudinal study identified in this review showed that retrospective reports of nightmares prior to a trauma predicted the later development of PTSD (Neylan et al., 2020).

A key question for clinicians is how and when to integrate treatments. For some patients treating PTSD will resolve their nightmares too, but for other patients the nightmares remain. For some patients it could be helpful for engagement in services to begin by treating nightmares, particularly if sleep disruption impacts on session attendance, and for others the nightmares will be severe enough that they should be the highest treatment priority. Research could inform this decision by investigating whether first stabilising sleep by treating nightmares enhances the efficacy of trauma focused CBT. Or whether treating PTSD in addition to sleep disturbance leads to a fuller recovery in nightmares.

Whilst nightmares are commonly considered in the context of anxiety, an under-recognised potential causal association highlighted by this review is between nightmares and depression. Treating nightmares resulted in moderate improvements in depression. Indeed, the effects were similar to those found for PTSD and marginally larger than for anxiety, despite the majority of participant groups being either trauma-exposed or having PTSD. However, no trials treated nightmares in a group recruited on the basis of major depressive disorder, which necessitates caution in interpreting nightmares treatment effect sizes on depressive symptoms. Further investigation into the association is clearly warranted. There are plausible mechanistic links between nightmares and subsequent low mood. First, nightmares result in sleep loss (a symptom of insomnia), which is an established causal factor for low mood (Freeman et al., 2017; Henry et al., 2021). Second, it is possible that nightmares trigger or reinforce negative core-beliefs (e.g. ‘the nightmares mean that I'm broken’; ‘my nightmares mean I'm a bad person’) and that these appraisals underpin the low mood. If found to be true, this hypothesis implies that where nightmares occur, they impact upon a core psychological mechanism for depression. In order to assess this theorised mechanism, measures which assess cognitive appraisals of nightmares - and particularly beyond threat related appraisals - are needed. This could elucidate mechanistic links with a broader range of affective states, beyond fear. Depression would be an interesting test case. If mechanistic links are not supported, it could be the case that participants simply feel happier after an improvement in their nightmares and therefore that treating nightmares may not target core psychological processes for depression.

Suicidal ideation can occur alongside depression. Longitudinal studies provided robust evidence that nightmares are associated with an increase in later suicidal ideation, attempts and death by suicide. The association was supported by six out of seven of the studies conducted. In a group of inpatients who recently attempted suicide (i.e., an enriched group), frequent nightmares at baseline were associated with three times the odds of a repeat suicide attempt over two years (Sjöström et al., 2009). Indeed, half of those experiencing frequent nightmares subsequently re-attempted suicide, compared with a third of those who did not have nightmares. The relationship between nightmares and suicide outcomes is partly mediated by negative affect (Liu et al., 2020; Sjöström et al., 2009). Defeat, entrapment and hopelessness are additional viable mechanisms that have been identified in a cross-sectional study, but require further testing (Littlewood, Gooding, Panagioti, & Kyle, 2016). Given the degree of good quality longitudinal data, the scarcity of trials treating nightmares and assessing suicidal ideation is a notable omission from the evidence base. Initial evidence from two small pilot trials indicates that treating nightmares may actually prevent recovery in suicidal ideation (Pruiksma et al., 2020; Sheaves et al., 2019). However both trials were small and suffered floor effects for this outcome. It remains possible that nightmares are a risk indicator for suicidal ideation, but not a causal factor in its occurrence. It cannot be ruled out for example that the relationship is underpinned by a shared cause. This clearly requires further investigation.

It is possible that nightmares are not directly associated with all psychiatric symptoms, but rather there is a more limited number of direct relationships and others are indirect. Given the role of REM sleep in fear extinction learning, it is plausible that there are direct reciprocal relationships with nightmares and threat-based disorders. However the majority of studies assessing other psychiatric symptoms did not explicitly control for anxiety. There is preliminary evidence that the longitudinal relationships between nightmares and both psychotic experiences (Fisher et al., 2014; Thompson et al., 2015) and suicide outcomes (Sandman et al., 2017; Sjöström et al., 2009) hold whilst controlling for PTSD or experiences of trauma. It is unclear however whether the reported relationship between nightmares and depression is secondary to the development of anxiety. Mediation analysis of a nightmare treatment trial could assess whether changes in outcomes such as depression occur via changes in anxiety severity.

There are notable gaps in the literature. Whilst nightmares occur in at least half of people experiencing psychosis and dissociative disorders there have been no causal tests of the hypothesis that nightmares result from these psychiatric symptoms. Similarly, nightmares occur in around half of those with borderline personality disorder, but there have been no controlled trials testing the effects of treating nightmares in this group. Nightmares have not been assessed in any longitudinal or manipulation studies in relation to bipolar disorder or eating disorders, and there is very limited literature on individual anxiety disorders. There have also been no tests of the impact of treating persistent and troubling nightmares on psychiatric symptoms in children. 7–11% of children report ‘often’ experiencing nightmares (Wiechers et al., 2011) and 1 in 5 regularly experience them (Fisher et al., 2014). If the promising reductions in depression, anxiety and PTSD following treatment of nightmares in adulthood also hold in child populations, treating nightmares could provide a preventive treatment option for the development of depression and anxiety disorders. A first step could be to ascertain sub-groups of children who are most vulnerable to the negative psychiatric sequalae of nightmares. Given that insomnia is a potential causal factor in the occurrence of a range of mental health problems (Freeman et al., 2017; Freeman et al., 2020), future studies are needed to assess whether the improvement in psychiatric symptoms following treatment of nightmares is mediated (and if so to what extent) by an improvement in insomnia.

There were many methodological limitations to the research reviewed. First, many studies relied on unvalidated measures of nightmares or single items taken from PTSD assessments. The development of psychometrically robust nightmare measures - particularly those that distinguish between trauma-related and idiopathic nightmares - is a priority for the area. These would benefit from being developed with lived-experience expertise, and a range of psychometric assessments completed (e.g. test-retest reliability and assessing measurement invariance). Second, participants in clinical trials were often not selected on the basis of co-occurring psychiatric symptoms, particularly so for depression and other anxiety disorders. It is therefore unclear whether findings generalise to clinical presentations of these disorders. Third, many trials were not powered to detect change in psychiatric outcomes other than nightmares. Now that the efficacy of treatments for nightmares is established (Casement & Swanson, 2012; Hansen et al., 2013), adequately powered trials should be designed in clinical groups to establish the additional effects on psychiatric symptoms. Fourth, half of the longitudinal studies did not control for the baseline level of the outcome, meaning that a more severe outcome may be reflective of more severe baseline levels of that problem. Lastly, whilst there are examples of trials which report consistent medication use across assessment time points (Pruiksma et al., 2020; Sheaves et al., 2019), there is limited reporting of medication data in several trials, which could be a confound. Whilst it is difficult to control completely for the effect of medication given that many patients take more than one medication, with potentially interacting effects, and there are ethical problems with limiting necessary medication changes within the course of a trial, measuring and reporting key groups of medications (e.g. using the defined daily dose approach (Norwegian Institute of Public Health, 2016)) may mitigate the risk of confounding to some degree.

There are several clinically important avenues for furthering the investigation of nightmares and their relation to psychiatric symptoms: first, qualitative studies to learn from the lived experience of co-occurring nightmares and suicidal ideation could develop clearer hypotheses about whether they are causally related or an epiphenomenon; second, a definitive test of treating nightmares in patients diagnosed with depression is warranted; third, given the high prevalence of nightmares in those with dissociative disorders and borderline personality disorder, testing the effects of treating nightmares is warranted in these clinical groups. and lastly, integrating assessments of nightmares into trials testing treatments for other disorders would allow an assessment of the traditional view that nightmares improve following resolution of waking distress requires addressing.

Dreaming has been the subject of much historical interest from philosophers, clinicians and writers. There remains a contemporary lay intrigue into nightmares, their impact and how to overcome them but this review shows that there has been a remarkable lack of scientific scrutiny into nightmares and the relationship with mental health problems. The limited literature suggests that alleviating nightmares may be one route to ameliorating threat-based disorders, there are promising effects of treating nightmares on depressive symptoms which warrant further investigation, and there are many disorders which remain vastly under-studied in the context of nightmares, but where emotion regulation difficulties mean there is a plausible mechanistic link (dissociative disorders and borderline personality disorder for example). Learning from patient experience through qualitative studies could prove particularly informative for elucidating potential mechanistic links between nightmares and psychiatric symptoms, and understanding the degree of treatment priority patients give to nightmares. Irrespective of the effects on psychiatric disorders, when nightmares are distressing and impact on functioning, evidence-based treatments should be offered because they are a diagnosable mental health problem (American Psychiatric Association, 2013) which can be highly disruptive to the lives of those suffering from them. This review highlights that there are likely other benefits on mental health too. The first step to offering treatments is better identification of nightmares in clinical services.

## Role of the funding source

This work was supported by a 10.13039/100010269Wellcome Trust Strategic Award (098461/Z/12/Z) to the Oxford Sleep and Circadian Neuroscience Institute (SCNi), with DF as one of the grant holders. BS is funded by a HEE/NIHR clinical doctoral research fellowship (ICA-CDRF-2017-03-088). DF is an NIHR Senior Investigator. This publication presents independent research funded by HEE/NIHR. The views expressed are those of the authors and not necessarily those of the NHS, HEE, the NIHR, or the Department of Health and Social Care. The funding sources had no role in the study design, collection, analysis or interpretation of the data, writing the manuscript, or the decision to submit the paper for publication.

## Contributors

BS conceived of the review idea. BS and SR conducted the literature search and reviewed full text papers. BS drafted the manuscript and SR and DF edited versions for important intellectual content.

## Declaration of Competing Interest

The authors have no competing interests to declare.

## Data Availability

No data was used for the research described in the article.

## Appendix A Supplementary data

Supplementary materials: quality appraisal results for included studies Image 1
