# Supplementary material for: Nightmares and psychiatric symptoms: A systematic review of longitudinal, experimental, and clinical trial studies
Source: Clin Psychol Rev. 2023 Mar;100:102241. doi: 10.1016/j.cpr.2022.102241 (PMC10933816; doi:10.1016/j.cpr.2022.102241)
Supplement: Supplementary file 1 — Supplementary materials: quality appraisal results for included studies [file mmc1.docx]

Table 1. Quality appraisal results for Controlled trials treating nightmares and assessing the impact on psychiatric symptoms

| **Citation** | **Was the study designed with the primary aim to assess the association between nightmares and a psychiatric symptom(s)?** | **Were the participant selection criteria appropriate for answering the review question?** | **Is the study powered to assess the relationship between nightmares and psychiatric symptoms? (i.e. is there a risk of a type II error?)** | **Key result for psychiatric symptoms** | **Is a validated measure of nightmares used?** | **Is a validated measure of psychiatric symptoms used?** | **Additional notes** |
| --- | --- | --- | --- | --- | --- | --- | --- |
| Ahmadpanah et al. (2014) | Not primary question. | Yes | No power calculation | Significant reductions in PTSD. | No | Yes |  |
| Burgess et al. (1998) | Not primary question. | No (psychiatric illness excluded). | No power calculation | Significant reductions in phobia and depression. | No | Yes |  |
| Davis & Wright (2007) | No distinction between primary and secondary research questions. Six sleep and psychiatric outcomes of interest, but participants recruited on the basis of nightmares (not psychiatric symptoms). | Yes. | No power calculation | Greater improvements in PTSD symptoms (*d*=.53) and depression (*d* = .59) following treatment versus waitlist control. | Yes | Yes |  |
| Davis et al. (2011) | Not primary question. | Yes | No power calculation | Significant improvements in depression (*d*=.37) and PTSD (*d*=.39). No significant reduction in dissociation (*d*=.11, *p*<.05). | Yes | Yes |  |
| Krakow et al (2000) | Not primary question. | Yes, and PTSD symptoms were an inclusion criteria. | No power calculation | The IRT group had significantly lower PTSD severity than control at 3 months. Within subject effect sizes: IRT *d*=1.20, waitlist *d=*.28. | Yes | Yes | In the intervention participants were requested to refrain from sharing a full disclosure of the traumatic event and only brief details relevant to nightmares were shared (e.g., being assaulted in the bedroom and hence keeping the lights on at night,), hence attempting to isolate nightmares from other PTSD symptoms. |
| Lancee et al. (2010) | Not primary question. | Mixed - those receiving treatment for PTSD or scoring high on PTSD assessment excluded. | Adequately powered for nightmares and depression, not anxiety or PTSD complaints. | Nightmares treated with IRT led to significant improvements in anxiety (*d*=.25) compared to waitlist control, but not depression (*d*=.26) or PTSD (*d*=0.11). Nightmares treated with IE led to significant reductions in depression (*d*=.56) but not anxiety (*d*=.13) or PTSD (*d*=0.09) compared with wait list. | Yes | Yes | Inclusion of attention control condition (recording of nightmares) in addition to wait list control. |
| Lancee et al. (2020) | No, designed to assess efficacy of self-help intervention for nightmares on nightmare severity. | Mixed - Depression was in the mild range at baseline and anxiety was in the healthy range. | Adequately powered for change in nightmares, but not depression or anxiety. | No significant Treatment × Time interactions were found for depressive symptoms (PHQ; *F*=1.68, *p*=.199, *d*=.30) or anxiety symptoms (HADS-A; *F*= 0.49, *p*=.488, *d*=0.19). | No | Yes |  |
| Pruiksma et al. (2020) | Designed as a pilot trial to estimate treatment effects.  No distinction between primary and secondary efficacy outcomes. Five sleep and psychiatric outcomes of interest, but participants recruited on the basis of nightmares (not psychiatric symptoms). | Experience of trauma was an inclusion criteria, but PTSD was not. Those who were at current suicide risk were excluded. | Underpowered (pilot study) | ERRT led to medium effect size improvements in depression (*d*=-0.51) and marginal improvements in PTSD (*d*=-0.12) compared with MCC at post-treatment. MCC led to marginally greater changes in suicidal ideation than ERRT (*d*=.16). The study was not powered to detect statistically significant changes of this magnitude. | Yes | Yes | Inclusion criteria: stable medication, and willing to refrain from new behavioural health or medication treatment for sleep or PTSD during the trial. |
| Raskind et al. (2007). | Not primary question. | Mixed, those with depression and with suicidal ideation were excluded. | No power calculation | A trend towards reduction in depressive symptoms (*p*=.08). No significant reduction in PTSD at 8 weeks. | No | Yes | - Methodological benefit of having placebo control. - Method of action of Prazosin is unclear, it may act upon PTSD too. |
| Raskind et al. (2013) | Not primary question. | Mixed – selection criteria appropriate for PTSD. Participants not selected for depression, though levels at baseline were in the clinical range. | No power calculation | Significant between group effect on PTSD favouring the prazosin group. | No | Yes | - Methodological benefit of having placebo control. - Method of action of Prazosin is unclear, it may act upon PTSD too. - The result remained significant after removal of the nightmare item from the PTSD assessment. |
| Sheaves et al. (2019) | Designed as a pilot trial to estimate treatment effects (nightmare severity was the primary efficacy outcome, not psychiatric symptoms). | Yes – clinical group experiencing psychosis. | Underpowered (pilot study) | Medium effect size reduction in paranoia (*d*=-0.6), no effect on hallucinations (*d*=0.1), reduction in dissociation (*d*=-0.8), mixed effects on negative affect. Suicidal ideation remained stable in CBT group but improved in TAU group (*d*=0.3 at post-treatment, *d*=0.7 at follow-up). | Yes | Yes | - Stable medication at point of recruitment. - Defined daily dose of key groups of medication reported at each assessment point. Broadly stable medication in both trial arms across the duration of the trial. |
| Taylor et al. (2008) | Designed to assess effect of Prazosin on objective sleep parameters and PTSD. | Yes – clinical group with PTSD. | No power calculation | Significant reduction in PTSD symptoms (*d*=0.79). | No | Yes | - Methodological benefit of having placebo control. - Method of action of Prazosin is unclear, it may act upon PTSD too. |
| Van Schagen et al. (2015) | Not primary question. | Yes | Adequately powered for change in nightmares, but not psychiatric symptoms. | Significant reduction in PTSD (*d*=.69) maintained at follow-up. Significant reduction in anxiety (*d*=.58), and depression (*d*=.55) at post-treatment, but fell short of significance at follow up. No significant reduction in agoraphobia. | Mixed | Yes | - Intervention aimed to avoid exposure to the trauma memory, hence attempting to isolate nightmares from other PTSD symptoms. - Item relating to ‘bad dreams’ removed from the PTSD assessment, suggesting that the treatment of nightmares impacts on other PTSD symptoms |

Table 2. Quality appraisal results for studies assessing the longitudinal association between nightmares and psychiatric symptoms

| **Citation** | **Was the study designed with the primary aim to assess the association between nightmares and a psychiatric symptom(s)?** | **Were the participant selection criteria appropriate for answering the review question?** | **Is the study powered to assess the relationship between nightmares and psychiatric symptoms? (i.e. is there a risk of a type II error?)** | **Main findings** | **Is a validated measure of nightmares used?** | **Is a validated measure of psychiatric symptoms used?** | **Is the baseline level of the outcome controlled for?** | **Additional notes** |
| --- | --- | --- | --- | --- | --- | --- | --- | --- |
| Bernert et al (2017) | Yes | Yes - Suicide attempt history and recent suicidal ideation. | Adequately powered | Nightmares associated with higher severity of suicidal ideation at 7 and 21 day follow up, after controlling for depression and baseline suicidal ideation. Insomnia and nightmares combined accounted for two thirds of the variance in suicidal ideation (R^2^=.69 at 7 days and .59 at 21 days). Nightmares were not a significant predictor of later mood variability. | Yes | Yes | Yes |  |
| Fisher et al. (2014) | Yes - use of existing dataset | Yes | No power calculation reported, though sample size is large | Frequent childhood nightmares predicted later psychotic experience at 12 years, after controlling for sex, family adversity, emotional or behavioural problems, IQ and potential neurological problems (OR = 1.16, p < .05). | No | Yes | No |  |
| Gerhart et al. (2014) | Yes | Yes | No power calculation reported, though sample size is large | Trauma related nightmares predicted later PTSD (β = .10, p <.05) and depression (β = .10, p <.05) but the reverse relationships were not significant. | No | Yes | Unclear |  |
| Greene et al (2015) | No | Unsure | No power calculation reported, though sample size is large | Parent reports of childhood nightmares at age 5 were not associated with depression over the past year at age 34 (OR=1.03; 95% CI: 0.84-1.56). | No | No | No |  |
| Hedström et al. (2021) | Yes - use of existing dataset | Yes | Unclear given the number of parameters controlled for in the nightmares analysis. Large sample size but suicide is an uncommon outcome. | Often or always having nightmares was associated with a significantly increased incidence of suicide. However, after adjustment (for seven covariates) statistical significance was lost. Among participants without a diagnosis of depression at baseline, the odds of depression during follow-up was higher among those who suffered from nightmares than among those who did not (OR= 1.35; 95% CI = 1.19-1.53). | No | No - medical records and death registers were used. | No |  |
| Kobayashi et al. (2013) | Yes (but pilot study) | Yes | Adequately powered for size of effect reported by male participants but may be underpowered for females. | Nightmares were associated with later PTSD in men (*r*=.53, *p*<.01) but the relationship was not significant in women (*r*=.30, *p*>.05). | Yes | Yes | No |  |
| Lereya et al. (2017) | Yes - use of existing dataset | Yes | No power calculation reported, though sample size is large | Having persistent childhood nightmares was associated with later borderline personality disorder symptoms in adolescence after controlling for sleep onset and maintenance problems and confounders including psychiatric diagnosis, emotional and behavioural problems, abuse and family adversity (adjusted OR= 1.62; 95% CI= 1.12-2.32). | No | Yes | No |  |
| Li et al. (2010) | Yes | Yes | No power calculation reported | Recurrent nightmares predicted later suicide attempts (OR= 8.17; 95% CI, 1.06-63.13). The addition of comorbid insomnia to nightmares increased the one-year incidence of suicide risk (OR= 17.08; 95% CI, 2.64-110.40). | No | No - based on medical records. | No |  |
| Li et al. (2012) | Yes | Yes | No power calculation reported | Participants experiencing at least one nightmare per week at baseline were less likely to be remitted from depression at follow-up compared with those without nightmares (with nightmares: 29.8% versus without: 47.3%, *p* < 0.01). | Yes | Yes | N/A |  |
| Li et al. (2016) | Yes | Yes – though results specific to suicide in schizophrenia. | No power calculation reported | Nightmare complaint alone did not predict the occurrence of suicide attempts, but the comorbidity of nightmares and insomnia was associated with the risk of suicide attempt over follow-up (adjusted HR = 11.10, p < 0.05). | No | No - based on medical records. | Yes |  |
| Liu et al. (2019) | Yes | Yes | No power calculation reported, though sample size is large | Frequent nightmares at baseline were significantly associated with suicide attempts and non-suicidal self-injury one year later. After adjusting for demographics, depression, impulsivity and prior suicide attempt, the association remained significant for suicide attempts (OR=1.96, 95% CI=1.15-3.33) and non-suicidal self-injury (OR=1.1.52; 95% CI=1.10-2.08). Adjustment for insomnia and sleep duration yielded almost no change. | No | No | Yes |  |
| Liu et al (2020) | Yes | Yes | No power calculation reported, though sample size is large | This is the same sample as Liu et al (2019). The log odds of endorsing suicidal thoughts, plans or attempts at follow-up were all significantly higher in those endorsing frequent nightmares at baseline. The relationship was partially mediated by depression. Adjusting for covariates and baseline suicidal behaviour reduced the size of effects. The same set of analysis assessing the relationship between nightmares distress and suicidal behaviour yielded mixed results. | No | Mixed | Yes | This is the same sample as Liu et al (2019). |
| Neylan et al. (2020) | Yes | Yes | No power calculation reported | Nightmares were a significant predictor of 2 week acute stress disorder (ASD) and 8 week PTSD, whilst controlling for retrospective reports of pre-trauma PTSD and peritraumatic symptoms (2 week ASD OR=1.3; 95% CI=1.0–1.5, 8 week PTSD OR=1.3; 95% CI=1.1-1.6). Nightmares predicted major depressive episode at 8 weeks (OR=1.3; 95% CI=1.0-1.6), whilst controlling for pre-trauma PTSD and depression. The association held whilst controlling for peritraumatic symptoms (OR=1.2; 95% CI=1.0-1.5). Nightmares were not significantly associated with depression at two weeks post-trauma (OR=1.2; 95% CI=1.0-1.5). | No | Yes | Yes |  |
| Pigeon et al. (2013) | Yes | Yes - though results specific to PTSD in military veterans with hazardous alcohol use. | No power calculation reported | Bothersome trauma related nightmares predict later PTSD severity. Of those endorsing nightmares at baseline, 41% developed PTSD, compared with 10% of those without. Trauma related nightmares were not associated with later depression. | No | Yes | No |  |
| Sandman et al (2017) | Yes - use of existing dataset | Yes | No power calculation reported, though sample size is very large | The unadjusted hazard ratio of suicide for persons who reported frequent nightmares compared with those who did not was 2.63 (*p*< .001), after adjustments this reduced to 1.84 (*p*= .010). The presence or absence of war veterans did not affect the association. | No | No - based on death register. | N/A |  |
| Shi et al. (2021) | Yes | Yes | No power calculation reported, though sample size is large | Endorsing frequent nightmares was associated with later suicidal ideation (OR= 1.69) and attempts (OR= 2.40) after controlling for a range of covariates including depression and baseline measure of the outcome. | No | No | Yes |  |
| Sjöström et al. (2009) | Yes | Yes – psychiatric inpatients admitted after a suicide attempt (i.e. an enriched group). | No power calculation reported | Having frequent nightmares at baseline predicted repeated suicide attempts (OR= 3.15; 95% CI= 1.51-6.57). Persistent nightmares further heightened the risk (OR= 5.20; 95% CI= 1.91-14.13). Results remained significant even after adjusting for sex, axis-I diagnosis, depression, anxiety, PTSD and anti-depressant drugs. Decrease in odds ratio (OR=2.28) after controlling for depression. | No | Mixed | N/A |  |
| Tanskanen et al. (2001) | Yes | Yes | No power calculation reported, though sample size is very large | Significant association between nightmares and later death by suicide. Among subjects having nightmares occasionally the adjusted relative risk of suicide was 57% higher, and among those reporting frequent nightmares 105% higher compared with subjects reporting no nightmares at all. | No | No - based on death register. | N/A |  |
| Thompson et al. (2015) | Yes | Yes | No power calculation reported, though sample size is large | Nightmares at 12 was associated with psychotic experiences age 18 when adjusting for IQ, family adversity, psychiatric disorders, depression, child abuse enuresis and psychotic experiences at 12 (OR = 1.62; 95% CI 1.19–2.20). The effect was larger for persistent psychotic experiences (at age 12 and 18 years, OR= 3.87; 95% CI: 2.30–6.51). | No | No | Yes |  |
| van Liempt et al. (2013) | Yes | Yes | No power calculation reported | Pre-deployment nightmares predicted later PTSD symptoms at six months post-deployment (OR= 2.99; 95% CI: 1.10-8.55), after controlling for pre-deployment PTSD, early trauma, mood and anxiety. | No | Yes | Yes | Nightmares assessed in military personnel prior to being deployed (i.e., before potential trauma). |
| Wittmann et al. (2010) | Yes | Yes | No power calculation reported | Trauma related nightmares ten days post-accident predict later PTSD total score two months post-accident, but not depression. | No | Yes | Yes |  |
| Wong et al. (2011) | Yes | Yes – but results specific to children of parents who are alcoholics. | No power calculation reported | Nightmares at age 12-14 did not predict later suicidal ideation or self-harm when ‘trouble sleeping’ was included in the model. | No | No | Yes |  |

Table 3. Quality appraisal results for Controlled trials treating a psychiatric symptom and assessing the impact on nightmares

| **Citation** | **Was the study designed with the primary aim to assess the association between nightmares and a psychiatric symptom(s)?** | **Were the participant selection criteria appropriate for answering the review question?** | **Is the study powered to assess the relationship between nightmares and psychiatric symptoms? (i.e. is there a risk of a type II error?)** | **Key findings** | **Is a validated measure of nightmares used?** | **Is a validated measure of psychiatric symptoms used?** | **Additional notes** |
| --- | --- | --- | --- | --- | --- | --- | --- |
| Gutner et al. (2013) | No, secondary analysis of existing trial. | Yes | No power calculation reported | Large reductions in nightmares following treatment of PTSD using CPT or PE. | No | Yes | Did not use intention to treat analysis. |
| Silver et al. (1995) | No – evaluation of existing inpatient PTSD programs. | Yes | No power calculation reported | EMDR for PTSD led to a significant reduction in nightmares (*p*< 0.05) when compared to control. | No | No | Did not use intention to treat analysis. |
| Woodward et al. (2017) | No, secondary analysis of existing trial. | Yes | No power calculation reported | CT-PTSD led to significant large effect size reductions in nightmares when compared with emotion focused supportive therapy and waitlist control (partial η2 = 0.15-0.16). | No | Yes | Sleep was not directly targeted in the intervention. |

Table 4. Quality appraisal results for the longitudinal association between psychiatric symptoms and nightmares

| **Citation** | **Was the study designed with the primary aim to assess the association between nightmares and a psychiatric symptom(s)?** | **Were the participant selection criteria appropriate for answering the review question?** | **Is the study powered to assess the relationship between nightmares and psychiatric symptoms? (i.e. is there a risk of a type II error?)** | **Key findings** | **Is a validated measure of nightmares used?** | **Is a validated measure of psychiatric symptoms used?** | **Is the baseline level of the outcome controlled for?** | **Additional notes** |
| --- | --- | --- | --- | --- | --- | --- | --- | --- |
| Levin & Fireman (2002) | Yes | Yes | No power calculation reported | Individuals who reported ≥ 3 nightmares (frequent nightmares) across the 3 weeks had significantly higher baseline scores on OCD, paranoia, psychoticism, dissociation and schizotypy. Depression and anxiety were not significantly higher at baseline in those reporting frequent nightmares. | Mixed | Yes | No |  |
| Short et al. (2017) | Yes | Yes | No power calculation reported | Daytime PTSD symptoms predicted nightmares later that night after controlling for baseline PTSD. Baseline depression, daily anxiety and daily stressors did not predict subsequent nightmares. | Yes | Yes | Yes |  |
